# A chemically fueled supramolecular glue for self-healing gels†

**DOI:** 10.1039/d2sc03691f

**Published:** 2022-09-15

**Authors:** Jennifer Rodon-Fores, Michaela A. Würbser, Martin Kretschmer, Benedikt Rieß, Alexander M. Bergmann, Oliver Lieleg, Job Boekhoven

**Affiliations:** Department of Chemistry, Technical University of Munich Lichtenbergstraße 4 85748 Garching Germany job.boekhoven@tum.de; TUM School of Engineering and Design, Department for Materials Engineering, Technical University of Munich Boltzmannstr. 15 85748 Garching Germany; Center for Protein Assemblies (CPA) & Munich Institute of Biomedical Engineering (MIBE), Technical University of Munich Ernst-Otto-Fischer-Str. 8 85748 Garching Germany

## Abstract

Chemically fueled supramolecular materials offer unique properties that include spatial and temporal control and even the ability to self-heal. Indeed, a few studies have demonstrated the ability to self-heal, however, the underlying mechanisms remain unclear. Here, we designed a peptide that forms a fibrillar network upon chemical fueling. We were surprised that the hydrogel could self-heal despite the lack of dynamics in the fiber assembly and disassembly. We explain this behavior by a mechanism that involves the chemically fueled peptide molecules that cannot self-assemble due to the lack of nucleation sites. When the fibers are perturbed, new nucleation sites form that help the assembly resulting in the healing of the damaged network. Furthermore, we generalized the behavior for other peptides. We refer to this non-assembling, chemically-fueled peptide as a molecular glue. In future work, we aim to explore whether this self-healing mechanism applies to more complex structures, narrowing the gap between biological and synthetic self-assemblies.

## Abstract 2

Chemically fueled supramolecular materials offer unique properties like the ability to self-heal, yet, mechanisms remain elusive. Here, we demonstrate that chemically fueled gels heal because of a molecular glue-based mechanism.

## Graphical abstract

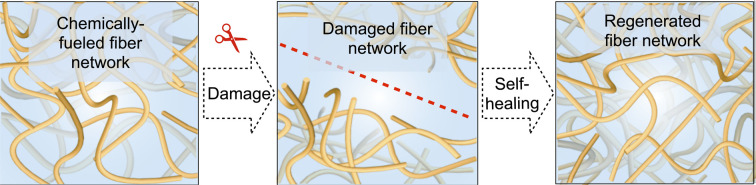

## Introduction

**Fig. 1:**
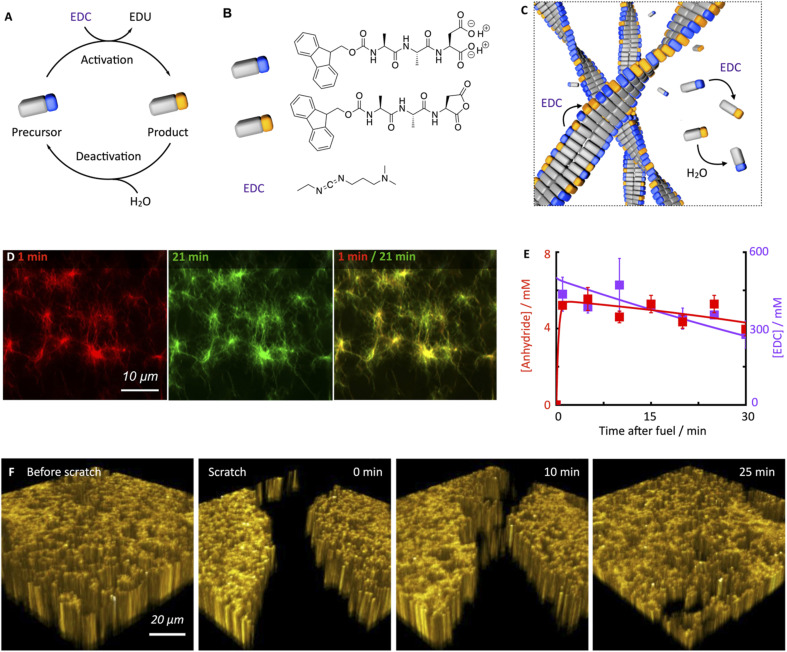
Chemically fueled self-assembly of Fmoc-AAD-OH into fibrillar assemblies in a pseudo-steady-state. (A and B) The chemical reaction cycle and peptide design. (C) Scheme of the fibers in pseudo-steady-state. Activation and deactivation occur at roughly equal rates both on the fibers and in the solution. Hardly any precursor disassembly takes place. (D) Confocal images of 10 mM Fmoc-AAD-OH fueled with 500 mM EDC, 1 minute and 21 minutes after application of fuel. The composite shows the lack of dynamic behavior. (E) Anhydride (red) and EDC (violet) concentration against time when 10 mM Fmoc-AAD-OH was fueled with 500 mM EDC. Markers represent HPLC data performed in triplicate; solid lines represent theoretical data, calculated using the kinetic model. (F) Confocal fluorescence microscopy in *x*/*y*/*z* dimension of 10 mM Fmoc-AAD-OH fueled with 500 mM EDC before performing a scratch and after the scratch performed 13 minutes after fuel addition, showing self-healing of the network over the course of 25 minutes.

Supramolecular materials are materials in which the building blocks are held together by non-covalent interactions.^1–6^ Recently, the focus has been on dynamic supramolecular materials that adapt to changes in their environment.^7–12^ A class of these dynamic materials is the chemically fueled supramolecular materials in which chemical reactions regulate self-assembly.^13–19^ In the chemical cycle, a precursor molecule reacts with a chemical fuel and becomes activated for self-assembly (*i.e*., the activation reaction, Fig. 1A). That activated state is transient and spontaneously reverts to the precursor. Thus, the building blocks for the assembly have a finite lifetime set by the activation and deactivation rates. Therefore, the properties of the supramolecular material are dictated by the kinetics of activation and deactivation and the rates of assembly and disassembly. The dynamic nature of these chemically fueled supramolecular materials offers exciting properties. For example, due to their fuel-dependent nature, these materials can be controlled over space and time.^16,20^ Specifically, if a finite amount of fuel is applied, a material emerges as long as fuel is available. This lifetime control has been explored in the context of self-abolishing hydrogels, transient emulsions, temporary nanoreactors, and others.^21–32^ When fuel is applied locally, the assemblies follow the gradients of fuel used in, *e.g.*, self-erasing inks.^33,34^ Finally, due to the constant activation and deactivation of self-assembling building blocks, the emerging supramolecular materials have been proposed to be self-healing.^35^ Indeed, biological chemically fueled assemblies, like the GTP-fueled microtubules, have been demonstrated to self-heal,^36,37^ and the underlying mechanisms are relatively well understood. In contrast, up to now, only one study indicates that chemically fueled synthetic fibers can self-heal.^38^ However, we still lack a fundamental understanding of the underlying self-healing mechanisms. Moreover, we do not know whether these principles are generalizable among chemically fueled assemblies.

In this work, we study how chemically fueled hydrogels can self-heal. We describe a small peptide that assembles into a fibrillar network at the expense of chemical fuel. Despite the fast dynamics of the reaction cycle, the fiber network is not dynamic in its assembly and disassembly. That means that building blocks are deactivated and reactivated in the fiber network without disassembling. Excitingly, after external damage, the network becomes dynamic, heals, and reinstates its static nature. Thus, chemical fuel is constantly burnt without any dynamic assembly. However, the mechanism of constantly burning fuel allows for healing in the case of damage.

## Results and discussion

**Fig. 2:**
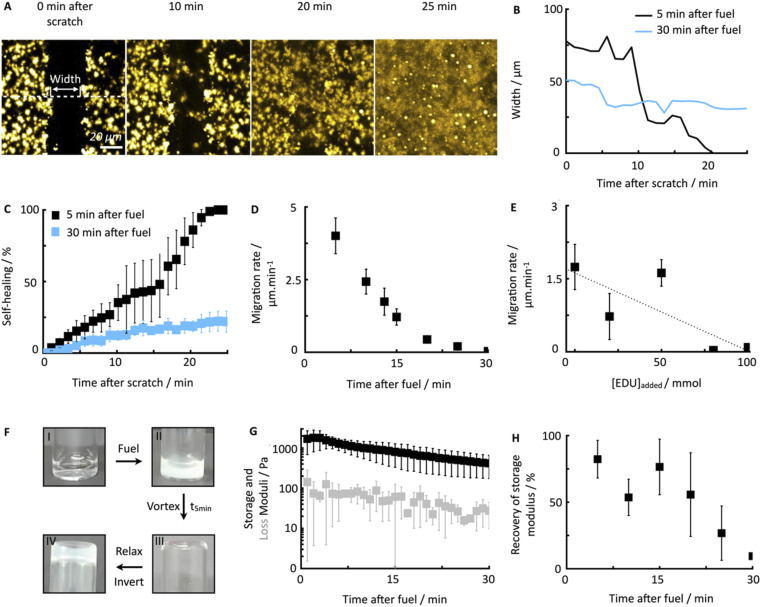
Self-healing behavior of chemically fueled Fmoc-AAD-OH in a pseudo-steady-state. (A) Confocal micrographs of damaged Fmoc-AAD-OH fibers at 5 minutes with collapsing width over the time of 25 minutes. For each scratch, a triplicate of widths was analyzed over the whole scratch size (each width being in a distance of roughly 40 μm from each other). (B) The width of a scratch as a function of time after damaging at 5 minutes (black) and 30 minutes (light blue). (C) Normalized self-healing behavior as a function of time after damaging at 5 minutes (black) and 30 minutes (light blue). Error bars represent 3 widths per scratch for an average of 3 scratches. (D) Migration rate of fiber assemblies over time of damage after fuel addition. Error bars represent 3 widths per scratch for an average of 3 scratches. (E) Migration rate over added EDU concentration, damaging at 13 minutes after fuel addition. Error bars represent 3 widths per scratch for an average of 3 scratches. (F) Vial inversion pictures of 10 mM Fmoc-AAD-OH without fuel (I), after the addition of 500 mM fuel (II), after vortex mixing 5 minutes after fuel addition (III), relaxing for 5 minutes and inverting (IV). (G) Storage and loss modulus over time of 10 mM Fmoc-AAD-OH fueled with 500 mM EDC. Error bars represent the average of 3 experiments. (H) Recovery of storage modulus of Fmoc-AAD-OH over different damage time after fuel addition. Error bars represent the average of 3 experiments.

The peptide we used in this study is based on the peptide Fmoc-AAD-OH in which Fmoc means fluorenylmethoxycarbonyl, A means Alanine, and D means aspartic acid (Fig. 1B). The peptide serves as a precursor in our chemical reaction cycle. In the cycle, we used *N*-(3-dimethylaminopropyl)-*N*′-ethylcarbodiimide (EDC) as fuel as previously published (Fig. 1B).^39,40^ In the activation reaction, the precursor reacts with EDC and is converted into its corresponding cyclic anhydride, *i.e.*, the product (Fig. 1B). In the aqueous environment, the precursor rapidly hydrolyzes back to the precursor state, *i.e*., the deactivation reaction. Taken together, upon application of fuel, a transient product emerges that is sustained until all fuel is depleted. Due to the loss of the two anionic carboxylates upon activation, the product can self-assemble into fibers driven by the Fmoc-group induced π-stacking and hydrogen-bond formation (Fig. 1C). Indeed, the peptide precursor was dissolved at a 10 mM concentration in an aqueous 200 mM MES buffer at pH 6, and no evidence of self-assembly was observed (see ESI, Fig. 1†). After applying 500 mM EDC, confocal microscopy revealed that a dense fibrillar network had formed (Fig. 1D and ESI, Fig. 2†).

We applied a significant excess of chemical fuel to ensure that the fibrillar network remained in a pseudo-steady-state, *i.e.*, that the activation and deactivation reaction rates were roughly equal (Fig. 1E). A pseudo-steady-state ensures that the network is not growing or collapsing during the analysis, which would not allow for an analysis of the self-healing behavior. By high-performance liquid chromatography (HPLC), we measured whether the 500 mM fuel was sufficient to keep the precursor and product in a pseudo-steady-state (Fig. 1E). Thus, at selected time intervals, we quenched a sample using a previously published method.^41^ We found that the product concentration was roughly 5.8 mM and remained relatively constant for the first 30 minutes. In this time frame, we also observed a high EDC consumption. Thus, the product remained in a pseudo-steady state due to the sufficiently high excess of fuel. We used a kinetic model that we previously wrote to predict the pseudo-steady-state kinetics and the consumption of fuel (Fig. 1E, ESI† supporting notes for a description of the model). The kinetic model predicts the concentrations of all species involved in the reaction cycle for each second by using five differential equations. From the kinetic model, we could calculate a half-life of the product of 46 seconds.

Despite the short half-life of the product, we were surprised to find that the fiber network is not dynamic in the sense that it does not assemble or disassemble (Fig. 1D). We can visualize the lack of dynamics by overlaying two micrographs of a 20 minute interval. When the two micrographs are overlaid, it is apparent that hardly any fibers have been newly formed or old ones disassembled. In other words, even though the product only has a half-life of 46 seconds, fiber disassembly hardly occurs.

We hypothesize that either the product assembles and does not deactivate after assembly resulting in a static network, or, the product is deactivated in the fiber and does not disassemble before its reactivation in the fiber, which also results in a static fiber network. In line with previous work on similar peptides,^40,42^ a ^1^H-NMR-study showed that most of the peptides, be it in the precursor or product state, reside in the assembled state (*vide infra*). Thus, the precursor remains kinetically trapped within the fibers after deactivation and is reactivated before it can disassemble (Fig. 1C). Taken together, our hydrogel can persist for roughly an hour in a pseudo-steady state but, surprisingly, does not display any dynamics of assembly or disassembly.

**Fig. 3:**
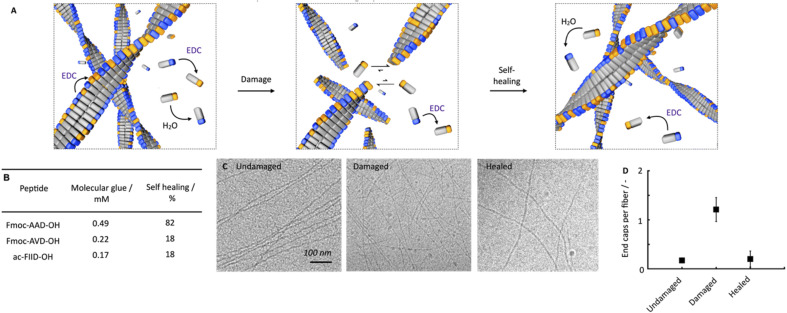
Self-healing mechanism of chemically fueled Fmoc-AAD-OH in a pseudo-steady-state. (A) Schematic depiction of the proposed self-healing mechanism. (B) Critical aggregation concentration of molecular glue and self-healing (rheological) at *t* = 5 minutes of Fmoc-AAD-OH, Fmoc-AVD-OH, and ac-FIID-OH. (C) Cryo-TEM micrographs of 10 mM Fmoc-AAD-OH fueled with 10 mM EDC without ultrasonication at *t* = 1 minute after fuel addition (undamaged), ultrasonication at *t* = 1 minute after fuel addition (damaged), 25 min after ultrasonication at 1 minute after fuel addition (healed). (D) Ratio of the number of end caps over the number of fibers per 1 μm^2^ at *t* = 1 minute after fuel addition without damaging (undamaged), damaging at *t* = 1 minute after fuel addition (damaged), 25 min after damaging at *t* = 1 min after fuel addition (healed). All experiments were performed in triplicate.

Next, we investigated the ability of this fiber network to self-heal by confocal fluorescence microscopy. Inspired by a cell wound healing assay which is based on manually drawing a scratch with a pipette tip through a line of epidermal cells,^43,44^ we developed a micromanipulator that can draw a scratch through our hydrogel with the tip of a needle (see Methods and ESI, Fig. 3†). In the assay, a hydrogel is made in an incubator chamber with a microscopy glass bottom window such that the hydrogel can be imaged by confocal microscopy. The micromanipulator drags a needle over the microscopy glass, such that it draws a trench through the gel in which the gel is damaged. Importantly, the hydrogel and the trench of damaged hydrogel remain hydrated such that no drying effects play a role in the healing process. The micromanipulator combined with an Arduino microcomputer allowed for automation and more reproducible scratching than manual perturbation. Specifically, we guaranteed an average scratch size of 54.9 μm (±12.4 μm). We used our micromanipulator on a fiber network in pseudo-steady-state (10 mM Fmoc-AAD-OH with 500 mM EDC) and drew a scratch 13 minutes after the fuel was added. Immediately after the scratch, we imaged the fiber network in three dimensions as a function of time (Fig. 1F). We were excited to observe that the scratch was, in fact, healing and that there was no evidence of the damage after roughly 30 minutes.

To quantify the healing, we repeated these experiments (Fig. 2A) and determined the width of the scratch (*W*, see Methods) in Fiji as a function of time after the scratch (Fig. 2B and see Methods). We found that *W* decreased gradually over 25 minutes until it reached zero, *i.e*., until the scratch was no longer visible (Fig. 2B). From this data, we concluded that the scratch heals to 100% over 25 minutes when a scratch is drawn 5 minutes after fuel addition. On another sample, we drew a scratch 30 minutes after adding fuel and found that the healing was much slower and incomplete. To compare the datasets, we calculated the degree of healing by measuring the amount healed (*W*_*t*=0_ − *W*) and normalized it towards the size of the initial width (*W*_*t*=0_) following eqn (1) (see Methods). Normalizing the size of the initial width allowed us to average multiple scratches (Fig. 2C). It should be noted that the fluorescence intensity in the micrographs decreased somewhat over the process of the healing (*e.g.*, Fig. 2A), which could be related to a morphological transition of the network after damaging it.

To confirm that the self-healing was an active behavior, we performed similar experiments with Fmoc-AAD-OH, which was acidified, *i.e*., a fiber network that was not maintained by a chemical reaction cycle but instead close to equilibrium. After the scratch, these fiber networks remained static without evidence of self-healing (see ESI, Fig. 4†). This data shows that chemically fueled fiber networks can heal and that the time of damage in the cycle plays a significant role in the ability of the network to heal.

To further understand the lack of healing, we also measured the average healing rate over 25 minutes after the scratch, *i.e*., the migration rate of the fiber front (Fig. 2D, see Methods eqn (2)). Again, we found that the healing was fast early in the cycle, but samples that were perturbed later in the cycle suffered from a slow healing process. The lack of healing later in the cycle is surprising, given that the reaction cycle is in a pseudo-steady state, *i.e*., the concentration of product and fuel did not drastically decrease in this time window. We hypothesized that the accumulation of the EDU waste caused the slowing of the healing.^45^ Thus, we measured the migration rate 13 minutes after adding fuel to the fibrillar networks with additional EDU (Fig. 2E). We found a peculiar relation between the migration rate and the EDU amount present in the system by adding different EDU concentrations before the fuel addition (Fig. 2E). With a concentration of 80 mM EDU or greater, no healing was observed. From these observations, we concluded that the active fiber networks are self-healing but accumulated waste severely hinders it.

Furthermore, we confirmed the ability of the fiber networks to self-heal on a macroscopic level. A vial inversion method visualized the ability of the hydrogel to recover and become self-supporting after damaging it on a vortex mixer for 20 seconds (Fig. 2F). Plate–plate rheology showed that after fueling with 500 mM of EDC, a hydrogel with a 1.4 kPa strength is formed that slowly loses stiffness as a function of time (Fig. 2G). After the vortex mixing, the damaged (liquified) gel converted back into a self-supporting hydrogel over the course of five minutes. In line with the previous data, we found that the gel could not recover when the damage was inflicted late, *i.e.*, after 30 minutes after adding fuel (ESI, Fig. 5†).

To quantify the recovery of the storage modulus, we performed the same vortexing experiment but analyzed the damaged gel with the rheometer. In line with the vial inversion tests, we found that recovery depended on the cycle time (Fig. 2H). Specifically, after 5 minutes in the reaction cycle, the gel without perturbation had a storage modulus of 1.4 kPa. A gel damaged on the vortex mixer after 5 minutes in the cycle had a strength of 0.7 kPa and recovered to 1.2 kPa over the course of 5 minutes (ESI, Fig. 6†). Thus, the gel was able to recover 82% of the original storage modulus. In line with the microscopy data, a gel damaged after 30 minutes in the cycle could only recover 9.4% of its initial storage modulus. So indeed, our chemically fueled Fmoc-AAD-OH assemblies show self-healing characteristics on a microscopic and macroscopic level, despite being limited due to the eventually accumulating EDU concentration.

We hypothesize that this self-healing behavior of the network, despite the lack of self-assembly and disassembly dynamics in the networks, originates from two effects (Fig. 3A). On the one hand, peptides are kinetically trapped in the fibers and activation, deactivation and reactivation take place on the fibers without disassembly. That would explain the lack of dynamics. On the other hand, part of the peptides is activated and deactivated in solution but cannot assemble because of a lack of endcaps of fiber to adhere to. After damaging the gel, the number of endcaps increases, and the healing process starts.

To confirm this hypothesis, we tested how much of the peptide is trapped in the fibers and how much of the peptide remains in the solution. We also tested how many endcaps become available during the damaging process. By ^1^H-NMR, we quantified that roughly 0.7 mM of the peptide remained in the solution in the first minutes. We assume that non-assembled peptides are NMR-active and visible whereas the assembled ones are not (ESI, Fig. 7A†). In other words, most peptide is assembled in the fibers. Combined with the HPLC data, which showed that there was roughly 5 mM precursor available under these conditions, this implies that most of the precursor is in the fibers. Thereby, we confirmed that the fibers consist of a co-assembly of anhydride product and precursor. Thus, there is a population of peptides that remains kinetically trapped in the fibers (roughly 9.3 mM), and there is a population of peptides in the solution that is continuously activated and deactivated (roughly 0.7 mM). We assume that the anhydride in the solution cannot assemble with the fibers because, on the one hand, its concentration is too low to nucleate into new fibers. On the other hand, the density of the endcaps is too low to add on existing fibers. In other words, the reaction cycle continuously converts chemical energy, but no new molecules assemble or disassemble. When a lower amount of fuel was added (100 mM, ESI, Fig. 7B†), similar kinetic trapping of the precursor in the fibers was observed. Even after all 100 mM fuel was depleted, disassembly had not completely taken place.

We established that peptides remain kinetically trapped. Next, we wondered how much of the peptide remains in solution. Thus, we determined the critical aggregation concentration of Fmoc-AAD-OH with 500 mM EDC. We added increasing amounts of Fmoc-AAD-OH to a solution of 500 mM EDC and measured the scattering rate by DLS (ESI, Fig. 8†). The amount of Fmoc-AAD-OH needed to find the first evidence of assemblies was 0.7 mM. We then used the kinetic model to calculate the amount of anhydride in that experiment to be around 0.5 mM. Thus, we conclude that the anhydride's critical aggregation concentration (CAC) of Fmoc-AAD-OH is ∼0.5 mM (Fig. 3B). From this surprisingly high number, we assume that, in our gels, most anhydride resides in the fibers. In contrast, roughly 0.5 mM anhydride is in the solution phase but cannot assemble due to its low concentration and the low number of endcaps. We refer to the anhydride in solution as the “molecular glue”. The scenario changes when the gel is destroyed (Fig. 3A, center). We expect that the number of endcaps drastically increases due to the damage. These endcaps are now available for the molecular glue to grow new fibers and aid healing.

We further supported the molecular glue hypothesis by cryo-TEM and counted the endcaps per fiber in 1 μm^2^ (Fig. 3C). The high gel stiffness made obtaining images challenging by cryo-TEM, so we changed the conditions to 10 mM Fmoc-AAD-OH fueled with 10 mM EDC. We normalized the number of end caps by the total number of fibers and found that the number of endcaps increased roughly 6-fold after ultrasonication. Moreover, after 25 minutes, the endcaps had drastically decreased again, further corroborating the self-healing through growing on endcaps (Fig. 3D). The observation that mild ruptures increase the chain termini and affect the seeded fiber growth has been demonstrated by others too.^46,47^

To further confirm the molecular glue hypothesis, we prepared a similar peptide that is fluorescently labeled with an NBD label on an additional cysteine (Fmoc-AAC(NBD)D-OH). We added the NBD label because of its sensitivity to its surrounding environment, thus allowing us to measure whether it is co-assembled or in solution. We prepared a gel of 10 mM Fmoc-AAD-OH fueled with 500 mM EDC and added a mixture of Fmoc-AAD-OH and Fmoc-AAC(NBD)D-OH on top in a ratio of 10 : 1 (ESI, Fig. 9†). We found that the fluorescence signal did not increase compared to a blank experiment of Fmoc-AAC(NBD)D-OH with fuel which points to no dye incorporation into the network. In contrast, when the dye was present from the beginning of the cycle, the fluorescence was very high, demonstrating that our NBD peptide indeed was incorporated. Finally, when adding the Fmoc-AAC(NBD)D-OH just after damage by vortexing or ultrasonication, the fluorescence signal increased drastically.

We received similar results on a microscopic level. By confocal fluorescence microscopy with the fluorescently tagged peptide and Nile Red, we imaged three samples, *i.e.*, an undamaged sample subjected to the fluorescently tagged peptide, a sample to which we added the fluorescently tagged peptide and then sonicated it, and a sample damaged with a needle loaded with fluorescently tagged peptide. Without damage, there was no evidence of any fluorescence intensity of the NBD-labeled peptide, demonstrating no incorporation (ESI, Fig. 10A†). In contrast, we observed homogeneous incorporation of the labeled peptide if the Fmoc-AAC(NBD)D-OH mixture was added on top of a gel and damaged by ultrasonication (ESI, Fig. 10B†). Lastly, we performed a scratch on a Fmoc-AAD-OH gel 13 minutes after fuel addition, applying a needle loaded with 1 μL of a Fmoc-AAD-OH/Fmoc-AAC(NBD)D-OH solution (ESI, Fig. 10C†). By this, we envisioned to provide the labeled peptide directly at the damaged front to track the spatial re-organization of the fibers. Over the migration time of 25 minutes, we observed a substantial increase in the intensity of the labeled peptide, specifically at the scratched site. These experiments further support our hypothesis that new building blocks are incorporated into the network only after damage as molecular glue aids the healing process.

To understand better the mechanism of self-healing, we tested two more chemically fueled, fiber-forming peptides, *i.e.*, Fmoc-AVD-OH and ac-FIID-OH.^40,48^ We chose Fmoc-AVD-OH due to its chemically structural similarity. Due to the more hydrophobic valine amino acid compared to alanine, it has a lower critical aggregation concentration, and we calculated the concentration of molecular glue to be also lower at 0.22 mM (Fig. 3B and ESI, Fig. 11†). On the other hand, ac-FIID-OH is structurally more different than Fmoc-AAD-OH. Yet, it also forms a hydrogel in response to chemical fuel and has a similar critical aggregation concentration. Its concentration of molecular glue is low at 0.17 mM (ESI, Fig. 11†). Cryo-TEM of these peptides demonstrated that they indeed form fibers (ESI, Fig. 12†).

By quantifying the recovery of storage modulus on the rheometer, we found that the self-healing ability of the other two fiber formers was lower than Fmoc-AAD-OH (ESI, Fig. 13†). Specifically, Fmoc-AVD-OH and ac-FIID-OH only showed a recovery of storage modulus of roughly 18%, 5 minutes after the application of fuel. These findings further highlight the importance of molecular glue for the healing of the hydrogels, *i.e.*, if the product has poor solubility, not much molecular glue is available, and healing is poor.

## Conclusions

We found that chemically fueled fibers can self-heal even though they are not dynamic. We explain this surprising finding by the concept of a chemically fueled molecular glue. Briefly, most peptides are assembled and will not disassemble upon deactivation, which explains the lack of dynamics. Some peptide remains in solution and is continuously activated and deactivated but cannot assemble into fibers due to their low concentration and the lack of nucleation sites to adhere to. The damaging of the network creates numerous new nucleation sites on which this molecular glue can self-assemble. The peptide's critical aggregation concentration is related to the concentration of molecular glue. Thus, the higher the critical aggregation concentration, the greater the ability to self-heal. It should be noted that the term molecular glue refers to a glue that can only heal its fibrillar network. Furthermore, we found that the behavior was generalizable for other peptides and confirmed that the system's CAC indeed notably influenced the self-healing nature. In future work, we aim to expand our understanding further and adapt the mechanism to more complex peptide structures.

## Materials and methods

### Materials

We purchased benzylamine (BA, 99%), deuterated dimethyl oxide (DMSO-*D*_6_, 100%, 99.96 atom % D), deuterium oxide (D_2_O, 99.96 atom % D), 1-ethyl-3(3-dimethylaminopropyl)carbodiimide hydrochloride (EDC·HCl, 99%), *N*,*N*′-diisopropyl-carbodiimide (DIC, 99%), 37 wt%. hydrochloric acid (HCl, MQ 200), 2-(*N*-morpho-lino)ethane sulfonic acid (MES, 99.5%), Nile Red (MQ 100), piperidine (99%), Fmoc-D(O*t*Bu)-wang resin (100–200 Mesh size, loading 0.67 mmol g^−1^), protected amino acids (Fmoc-A-OH (95%), Fmoc-V-OH (>98%), Ac-F-OH (98%), Fmoc-I-OH (98%)), sodium hydroxide (NaOH, >98%), *N*,*N*-dimethylformamide (DMF, 99.8%), diethyl ether (Et_2_O, >99%) trifluoroacetic acid (TFA, 99%), triisopropylsilane (TIPS, 99%), *N*-ethyldiisopropylamine (99%, DIEA), 4-chloro-7-nitrobenzo-furazan (NBD-Cl, 98%), β-mercaptoethanol (>99%) and 4-methyl-morpholine (NMM, 99%) from Sigma-Aldrich. Hydroquinone (99%) was purchased from Thermo Fisher Scientific and oxyma (97%) from Novabiochem. High-performance liquid chromatography (HPLC) grade acetonitrile (ACN) was purchased from VWR. All chemicals were used without any further purification unless stated differently.

### Synthesis of peptide precursor acids

Peptide precursor acids were prepared on a 0.250 mmol scale using a CEM Liberty microwave-assisted peptide-synthesizer and the Liberty Blue Application Software (Copyright CEM Corporation 2015, Version: 1.45.5794.20265). We used the preloaded wang resin with Fmoc-D(O*t*Bu)-OH as the first coupling step. The resin is pre-swollen outside the synthesizer for 15 min. Ahead of the amino acid coupling, the *N*-terminal Fmoc-protecting group was removed by adding a 20% v/v solution of piperidine (2 × 20 mL) in DMF. After heating in the microwave (1 minute, 90 °C), the mixture was washed with DMF (2 × 10 mL). Next, the coupling step was performed using 4.0 eq. of Fmoc-A-OH, Fmoc-V-OH, Fmoc-F-OH or Fmoc-I-OH in DMF (200 mM, 10 mL), 4 eq. of DIC (500 mM, 4 mL) and 4 eq. of oxyma (1000 mM, 2 mL). The mixture was subsequently heated in the microwave (2 minutes, 90 °C). The Fmoc-deprotection, washing, and the washing cycle was performed for each amino acid coupling. The peptide was cleaved from the resin by adding a mixture of 95% TFA (9.5 mL), 2.5% deionized water (0.25 mL) and 2.5% TIPS (0.25 mL). Afterwards, the solvent was removed under reduced pressure. The peptides were then precipitated out using diethyl ether. The crude product was isolated by centrifugation. The resulting peptides were dissolved in ±50% MeCN in H_2_O and purified by high-reversed-liquid-chromatography (RP-HPLC) (40% to 98% gradient, 0.1% TFA in H_2_O and MeCN). The collected fractions were freeze-dried and stored at −20 °C.

Fluorescent labelled peptide synthesis (Fmoc-AAC(NBD)D-OH): The anchoring of the first amino acid Fmoc-D(O*t*Bu) to the hydroxylic group of a wang resin was performed by the activated symmetric anhydride route. A solution (0.2 M) was prepared by dissolution of Fmoc-D(O*t*Bu)-OH (12.0 mmol) and DIC (6.0 mmol) in DMF at room temperature. The DIU side product was precipitated by cooling down the reaction mixture at −20 °C for 60 min and filtered out before being brought to contact with the resin. All subsequent couplings steps were performed with the microwave-assisted peptide synthesizer. As a first step, the symmetric anhydride solution was added to the pre-swollen wang resin (0.5 mmol) together with DMAP in catalytic amounts to drive the acetylation reaction. Afterwards, the *N*-terminal Fmoc protecting group was cleaved with piperidine solution at 20% v/v in DMF (2 × 20 mL) and Fmoc-C(Mmt)-OH and Fmoc-A-OH were added in the same way as described before.

The orthogonal deprotection of Mmt protecting group was performed by using a low percentage of TFA (2%) with TIPS in DCM (7 × 10 mL) stirred for 10 min. The deprotection progress could be followed by the yellow trityl cations formation. At the last wash step, the solution is then colourless. To prevent the formation of disulphide bond, β-mercaptoethanol with NMM were added to the solution as oxidizing agent. Then, the NBD-Cl was coupled to the cysteine. NBD-Cl (2.0 eq., 1.0 mmol) was added and followed by DIEA (1.0 eq. 0.5 mmol). The reaction is stirred at room temperature for 2 h in aluminium foil to prevent photobleaching of the dye. The cleavage of the peptide from the resin and its purification followed the same protocol as described earlier.

### Sample preparation

We dissolved the peptide precursor acids in 200 mM MES buffer resulting in a 13.3 mM stock solution, and adjusted the pH to pH 6.0. A 2000 mM EDC stock solution was always freshly prepared in MQ water, and the reaction cycle was started by adding EDC to the peptide precursor acid solution. A 1000 mM benzylamine stock solution in acetonitrile was prepared freshly ahead of the quenching experiment. All experiments were performed at 25 (±0.5) °C and stored at 8 and −20 °C.

### Quantification of the anhydride concentration

We prepared 75 μL of a 13.3 mM stock solution of the precursor acid into a screw cap HPLC vial with a micro insert and added the appropriate amount of fuel by adding 25 μL of a 2000 mM EDC stock in MQ water. The reaction cycle is then quenched at the respective time points with 100 μL of a 1000 mM BA stock solution in acetonitrile following a previously reported protocol.^41^ The BA reacts irreversibly with the anhydride groups and inhibits the reaction with the remaining EDC. The formed BA-peptide derivative was trackable by HPLC (220 nm) (ESI, Fig. 14–17†). This derivative concentration is equal to the formed anhydride concentration.

### Quantification of migration rate

We quantified the migration rate using a classical method for wound healing scratch assays.^43,44^ Herein, we damaged our fiber network using a 200 μm thin needle that was attached to a manipulator set-up (ESI, Fig. 3†). The scratch was performed on a 10 μL volumed hydrogel (10 mM Fmoc-AAD-OH fueled with 500 mM EDC, stained with 2.5 μM Nile Red), preformed in an incubation chamber (ibidi) suitable for imaging by confocal fluorescence spectroscopy without drying effects. The scratch was performed at the respective time by inserting the needle in 45° to the hydrogel layer and subsequently instructing the manipulator to perform the scratch. The healing process was immediately monitored in *x*/*y*/*z* dimension (184 μm × 184 μm with a *z*-stack of around 20 μm) over the migration time of 25 min. The analysis of the migration rate took place around 15 μm above the glass slide surface. Each scratch was performed in triplicate. We analyzed the scratch width collapse based on the manual distance measured by the software ImageJ*.* Herein, we took a width of the fibers front orthogonal to the scratch and compared the initial width at *t* = 0 minute to the one at *t* = 25 minutes. For each scratch, a triplicate of widths was analyzed over the whole scratch size (each width being in a distance of roughly 40 μm from each other). The healing in % was determined as follows:1
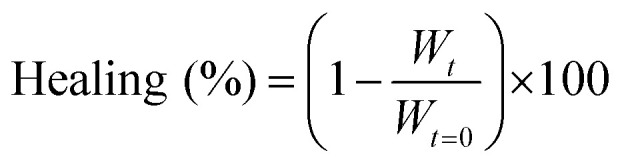


The migration rate was determined by applying eqn (2):2
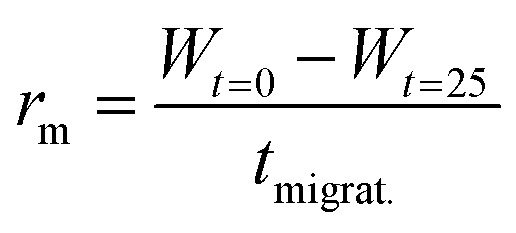
where *r*_m_ is the migration rate, *W* the width at different times *t* and *t*_migrat_ the time over which the network migration was studied (25 minutes).

### Quantification of peptide precursor in solution and co-assembly ratio

The amount of peptide precursor in solution and the co-assembly ratio of peptide precursor to anhydride product was investigated over time using ^1^H-Nuclear Resonance Spectroscopy (NMR) and analytical High-Performance Liquid Chromatography (HPLC). ^1^H-NMR spectroscopy was used to determine the free peptide precursor concentration in solution and the ratio of co-assembly of peptide precursor and product. We used an inner tube filled with an internal hydroquinone standard (50 mM dissolved in D_2_O) and recorded the spectra according to water suppression. Herein, we compared the integral of the Fmoc-protons at 7.20–7.50 ppm to the integral of the hydroquinone standard at 6.70 ppm to determine the free peptide precursor concentration in the solution. We confirmed the co-assembly ratio by assuming that self-assembled molecules do not show NMR-activity and remain silent, in contrast to non-assembled ones that are visible.^40^

In combination with the anhydride concentration that we obtained by HPLC, the peptide concentration in solution and the peptide precursor concentration was received as follows:3
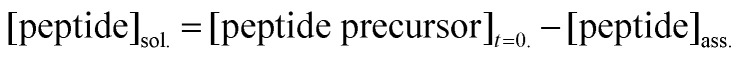


The amount of peptide precursor co-assembling in the fiber network was obtained according to:4
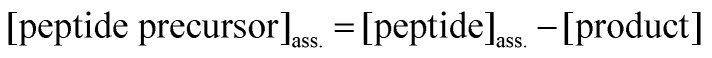
where [peptide]_sol._ represents the concentration of peptide in solution, [peptide precursor]_t=0_ the initial peptide precursor concentration, [peptide]_ass._ the concentration of peptide in the fiber assemblies, [peptide precursor]_ass._ the peptide precursor concentration in the fiber assemblies and [product] the concentration of anhydride product analyzed by HPLC. Using the so obtained peptide concentration in solution and our kinetic model, we could define the amount of anhydride product in solution that we define as molecular glue concentration.

### Spatial organization of Fmoc-AAC(NBD)D-OH

By confocal microscopy, we analyzed the spatial incorporation of the NBD-labeled peptide at the damaged network front. As a negative control (undamaged), we prepared 10 mM Fmoc-AAD-OH with 500 mM EDC (stained with 0.1 μM Nile Red) in the incubation chamber topped with 1 μL of a mixture (10 : 1 ratio) of Fmoc-AAD-OH and Fmoc-AAC(NBD)D-OH. We used an excitation at 488 nm for the NBD-labeled peptide and 552 nm for the Nile Red stained one. The gel was screened in *x*/*y*/*z*-dimension for 40 minutes.

Next, the damaged experiment was approached by preparing another Fmoc-AAD-OH gel in a vial topped with 1 μL of a mixture (10 : 1 ratio) of Fmoc-AAD-OH and Fmoc-AAC(NBD)D-OH. After 13 minutes the gel was ultrasonicated for 2 minutes, immediately transferred to the incubation chamber and screened as stated above.

Finally, the Fmoc-AAD-OH gel was prepared in the incubation chamber and scratched 13 minutes after fuel addition. The applied needle was topped with 1 μL of a mixture (10 : 1 ratio) of Fmoc-AAD-OH and Fmoc-AAC(NBD)D-OH, creating the direct contact of fiber damage sites and the NBD tagged peptide. All experiments were performed in triplicate.

### Quantification of amounts of end caps per fiber

The change in the number of end caps per fiber upon damage of the fiber network was determined using cryo-Transmission Electron Microscopy (TEM). Due to the gel's strength, we decreased the fuel concentration to 10 mM by adding 25 μL of a 40 mM EDC stock solution to a 75 μL peptide precursor acid stock solution of 13.3 mM in a 2 mL vial. The damaged sample was destroyed by ultrasonication of the sample for 20 seconds at *t* = 1 minute and immediately pipetted onto the prepared grid in the Vitrobot. The same sample was then pipetted on a second grid after 25 minutes to compare for the recovery of destroyed fibers. As a control, an undamaged sample with the same conditions was prepared for the same timepoints without ultrasonicating it. Per condition, six micrographs were used to determine the average end cap per fiber ratio. All experiments were performed in triplicate.

### High-performance liquid chromatography (HPLC)

We monitored the kinetics of the reaction cycle by analytical HPLC (Thermo Fisher Vanquish Duo UHPLC) with a Hypersil Gold 100 × 2.1 mm C18 column (3 μm pore size). We prepared 200 μL of the reaction mixture into a screw cap HPLC vial with micro inset following the sample preparation protocol described above. All samples were directly injected without any further dilution from the HPLC vial. We injected 2 μL for the detection of EDC, BA and for the detection of the peptide precursor acids. We used a UV/Vis detector at 220 nm and 254 nm for the quantification of all reagents. Calibration curves for the EDC (in MQ water), BA (in acetonitrile), peptide precursor acids (in MES buffer) were performed with the corresponding method in triplicate. Retention times and calibration values are given in ESI, Table 1.† We used the following method for the separation of the compounds:

HPLC method: H_2_O : ACN from 98 : 2 to 2 : 98 in 12 minutes, 2 : 98 for 1 minute, 2 : 98 to 98 : 2 in 1 minute and 98 : 2 for 1 minute resulting in a 15 minute long method.

HPLC: *R*_*t*_ (Fmoc-AAD-OH) = 9.7 min (see ESI, Fig. 15†).

HPLC: *R*_*t*_ (Fmoc-AVD-OH) = 11.1 min (see ESI, Fig. 16†).

HPLC: *R*_*t*_ (ac-FIID-OH) = 8.9 min (see ESI, Fig. 17†).

HPLC: *R*_*t*_ (Fmoc-AAC(NBD)D-OH) = 11.7 min (see ESI, Fig. 18†).

### Reversed-phase high-performance liquid chromatography (RP-HPLC)

The peptide precursor acids were purified on reversed-phase HPLC (Thermo Fisher, Hypersil Gold 250 × 20 mm, 5 μm pore size, C18 column) with a linear gradient of acetonitrile (ACN, 40 to 98%) and water with 0.1% trifluoroacetic acid. UV-Vis detection was conducted using 220 and 254 nm.

### Kinetic model

We used a Matlab kinetic model for the calculation of the concentrations of reactants. See ESI† supporting notes for a detailed discussion.

### Time-lapse photography and vial inversion

The vial inversion experiment was performed in a 2 mL HPLC vial and imaged with a high-definition camera. Time-lapse software was programmed to image the sample in a 30 second interval. We started the hydrogel formation by adding 125 μL of a 2000 mM EDC stock solution to 375 μL of 13.3 mM peptide precursor acid solution (pH 6, 200 mM MES). At the respective time, the hydrogel was destroyed using a vortex mixer for 20 seconds resulting in the liquification of the gel. After giving the gel time to relax for 5 minutes, the vial was inverted and imaged to demonstrate the gel's ability to self-heal. All experiments were performed in triplicate.

### Self-healing measure by rheology

We obtained the initial gelation morphology by adding 50 μL of a 2000 mM EDC stock solution to a 150 μL of a 13.3 mM peptide precursor acid in the rheometer. To ensure linear material response during the gelation process of the samples, the measurements were performed in torque-controlled mode (by applying small torques of 0.5 μNm and a constant oscillation frequency of 1 Hz). The storage and loss moduli were determined for 5 minutes, and one measurement point was recorded every 7.5 seconds. To determine the self-healing behavior of the peptides, the samples were prepared in a vial using the conditions stated above. At the respective time intervals, the gel was destroyed by a vortex mixer (20 seconds) and immediately transferred to the rheometer plate. The rheological measurement was conducted as described above to test for the mechanical recovery abilities of the sample over time.

### Confocal fluorescence microscopy

We imaged the behavior of the fiber assemblies on a microscopic level using a Leica DMi8 microscope, including a 63× water immersion objective. We prepared the hydrogels of a total reaction volume of 10 μL directly in incubation chambers (ibidi chamber). The assemblies were stained with 2.5 μM Nile Red. Samples were excited at 552 nm (488 nm in the case of Fmoc-AAC(NBD)-OH) and imaged at 577–650 nm.

### Fluorescence spectroscopy

Nile Red assay studies were performed on a Jasco FP-8300 fluorescence spectrophotometer with external temperature control (Jasco MCB-100). The fluorescence intensities were measured over time, each minute, at 635 nm with an excitation at 488 nm. The Fmoc-AAC(NBD)D-OH incorporation experiment was performed by preparing a hydrogel (135 μL of a 13.3 mM Fmoc-AAD-OH solution fueled with 45 μL of a 2000 mM EDC solution) in a vial. After 13 minutes, the gel was damaged (vortex mixed or ultrasonicated for 20 seconds) and pipetted to a 10 mm quartz cuvette from Precision Cells Inc. On top of the damaged gel, 20 μL of a 10 : 1 mixture of Fmoc-AAD-OH to Fmoc-AAC(NBD)D-OH was added. The increase in fluorescence intensity was observed over time. All experiments were performed in triplicate.

### Nuclear resonance spectroscopy (NMR)

NMR spectra were recorded on a Bruker AVIII-300, Bruker ARX 300 and AVIII-500-cryo spectrometer by Bruker Analytik at 25 °C and a frequency of 300/300/500 MHz, respectively. Chemical shifts *δ* are reported in ppm and are referred to the residual solvent peak of the used deuterated solvent (D_2_O (4.79 (^1^H))) and dmso-*D*_6_ (2.50 (^1^H) and 39.5 ppm (^13^C)). The abbreviation of the signal multiplets are stated as followed: s-singlet, d-doublet, t-triplet, q-quartet, m-multiplet. The coupling constant *J* referred to the average value in Hz and the coupling between two protons. The analysis of all received spectra was performed using MestReNova© software (Version 11.0.0.-17609).

^1^H-NMR ((Fmoc-AAD-OH) 300 MHz, dmso-*D*_6_): *δ* (ppm) = 12.61 (s, 2H, COO*H*), 8.10 (d, 1H, ^3^*J* = 8.0 Hz, CON*H*), 7.99 (d, 1H, ^3^*J* = 7.6 Hz, CON*H*), 7.89 (d, 2H, ^3^*J* = 7.5 Hz, C*H*_aryl_), 7.73 (dd, 2H, ^3^*J* = 6.3 Hz, C*H*_aryl_), 7.52 (d, 1H, ^3^*J* = 7.7 Hz, CON*H*), 7.37 (dt, 4H, ^3^*J* = 7.4 Hz, ^4^*J* = 1.2 Hz, C*H*_aryl_), 4.52 (dt, 1H, ^3^*J* = 7.9, 6.1 Hz, C*H*), 4.31–4.20 (m, 4H,CH, C*H*_2_), 4.06 (t, 1H, ^3^*J* = 7.2 Hz, C*H*), 2.67 (dd, 1H, ^2^*J* = 16.7 Hz, ^3^*J* = 5.8 Hz, C*H*_2_COOH), 2.59 (dd, 1H, ^2^*J* = 16.7 Hz, ^3^*J* = 6.6 Hz, C*H*_2_COOH), 1.23 (dd, 6H, ^3^*J* = 7.1 Hz, C*H*_3_) (see ESI, Fig. 19†).

^13^C-NMR ((Fmoc-AAD-OH) 250 MHz, dmso-*D*_6_): *δ* (ppm): 172.2, 171.9, 155.9, 144.1, 140.9, 127.9, 127.3, 125.6, 120.35, 65.8, 50.1, 48.7, 48.0, 46.8, 36.1, 18.5 (see ESI, Fig. 20†).

^1^H-NMR ((Fmoc-AVD-OH) 300 MHz, dmso-*D*_6_): *δ* (ppm) = 12.7 (s, 1H, COO*H*), 12.45 (s, 1H, COO*H*), 8.26 (d, 1H, ^3^*J* = 7.8 Hz, CON*H*), 7.90 (d, 2H, ^3^*J* = 7.6 Hz, C*H*_aryl_), 7.83 (d, 1H, ^3^*J* = 7.5 Hz, CON*H*), 7.73 (dd, 2H, ^3^*J* = 6.3 Hz, C*H*_aryl_), 7.59 (d, 1H, ^3^*J* = 7.7 Hz, CON*H*), 7.38 (dt, 4H, ^3^*J* =7.4 Hz, ^4^*J* = 1.5 Hz, C*H*_aryl_), 4.52 (dt, 1H, ^3^*J* = 7.8, 6.5 Hz, C*H*), 4.28–4.19 (m, 4H, CH, C*H*_2_), 4.15–4.09 (m, 1H, C*H*), 2.72–2.56 (m, 2H, C*H*_2_), 2.00–1.93 (m, 1H, C*H*), 1.20 (d, 3H, ^3^*J* = 7.1 Hz, C*H*_3_), 0.83 (dd, 6H, ^3^*J* = 7.1, 6.8 Hz, C*H*_3_) (see ESI, Fig. 21†).

^13^C-NMR ((Fmoc-AVD-OH) 250 MHz, dmso-*D*_6_): *δ* (ppm) = 172.8, 172.7, 172.1, 171.1, 156.1, 144.3, 141.2, 128.1, 127.6, 125.8, 120.6, 66.0, 57.4, 50.4, 49.0, 47.1, 36.3, 31.5, 19.6, 18.7, 18.3 (see ESI, Fig. 22†).

^1^H-NMR ((ac-FIID-OH) 300 MHz, dmso-*D*_6_): *δ* (ppm) = 12.53 (s, 2H, COO*H*), 8.19–8.12 (m, 3H, CON*H*),7.81 (d, 1H, ^3^*J* = 9.0, CON*H*), 7.25–7.20 (m, 4H, C*H*_aryl_), 7.19–7.15 (m, 1H, C*H*_aryl_), 4.65 (dd, 1H, ^3^*J* = 8.0, 5.9 Hz, C*H*), 4.51 (dd, 1H, ^3^*J* = 8.0, 5.9 Hz, C*H*), 4.18 (dd, 1H, ^3^*J* = 8.9, 7.3 Hz, C*H*), 2.91 (dd, ^3^*J* = 7.3, 5.7 Hz, C*H*), 2.75–2.65 (m, 2H, C*H*_2_), 2.55 (dd, 1H, ^3^*J* = 7.1, 5.1 Hz, C*H*), 1.74 (s, 3H, CH_3_), 1.70–1.63 (m, 2H, C*H*_2_COOH), 1.42 (dqd, 1H, ^2^*J* = 12.3 Hz, ^3^*J* = 7.3, 3.2 Hz, C*H*_2_), 1.23 (dqd, 1H, ^2^*J* = 12.4 Hz, ^3^*J* = 7.2, 3.3 Hz, C*H*_2_), 1.05 (dqd, 1H, ^2^*J* = 14.0 Hz, ^3^*J* = 8.9, 7.2 Hz, C*H*_2_), 0.92 (dqd, 1H, ^2^*J* = 14.1 Hz, ^3^*J* = 8.8, 7.1 Hz, C*H*_2_), 0.82–0.71 (m, 12H, C*H*_3_) (see ESI, Fig. 23†).

^13^C-NMR ((ac-FIID-OH) 250 MHz, dmso-*D*_6_): *δ* (ppm) = 127.7, 172.1, 171.6, 171.1, 169.4, 138.2, 129.7, 128.4, 126.7, 57.0, 54.3, 48.9, 38.7, 37.1, 36.7, 36.3, 24.5, 22.9, 15.8, 11.4 (see ESI, Fig. 24†).

^1^H-NMR ((Fmoc-AAC(NBD)D-OH) 300 MHz, dmso-*D*_6_): *δ* (ppm) = 12.83 (s, 1H, COO*H*), 12.50 (s, 1H, COO*H*), 8.56–8.49 (m, 2H, CON*H*), 8.35 (d, 1H, ^3^*J* = 8.2 Hz, C*H*_aryl_CNO_2_), 8.06 (d, 1H, ^3^*J* = 7.0 Hz, C*H*_aryl_CS), 7.87 (d, 2H, ^3^*J* = 7.8 Hz,N*H*), 7.71 (dd, 2H, ^3^*J* = 11.5, 7.0 Hz, C*H*_aryl_), 7.56 (dd, 2H, ^3^*J* = 8.4 Hz, C*H*_aryl_), 7.4 (td, 2H, ^3^*J* = 7.5 Hz, ^4^*J* = 2.6 Hz, C*H*_aryl_), 7.35–7.30 (m, 2H, C*H*_aryl_), 4.78–4.53 (m, 2H, C*H*_2_CHCH_aryl_), 4.26–4.18 (m, 3H, C*H*), 3.72–3.49 (m, 2H, C*H*_2_S), 2.73–2.67 (m, 2H, C*H*_2_COOH), 1.20 (d, 6H, ^3^*J* = 7.2 Hz, C*H*_3_CH) (see ESI, Fig. 25†).

### Dynamic light scattering (DLS)

The critical aggregation concentration (CAC) was determined on a Malvern Zetasizer Nano ZS using a laser wavelength of 633 nm and disposable cuvettes (PS), setting the method of 5 acquisition times of 20 seconds per measurement. A series of low peptide precursor acid concentration was fueled with 500 mM EDC (375 μL of × mM peptide precursor acid solution fueled with 125 μL of a 2000 mM EDC solution, prepared directly in the cuvette) to observe a significant increase in the scattering rate. All measurements were performed in triplicate.

### Rheology

We studied the gelation behavior of the peptide assemblies using a commercial shear rheometer (MCR 302, Anton Paar, Graz, Austria) and a plate–plate geometry (bottom plate: P-PTD200/80-I, Anton Paar, equipped with a *Ø* 55 mm polystyrene Petri-dish (VWR, Radnor, USA); *Ø* 25 mm steel measuring head: PP25, 79044, Anton Paar). For each measurement, the required sample volume was 180 μL as the plate separation was set to 0.3 mm. A solvent trap (a chamber containing a water-soaked sponge, covered with a lid) as well as a temperature control of the bottom plate (set to 21 °C) was employed for all measurements. All measurements were recorded in triplicate.

### Electron spray ionisation-mass spectrometry (ESI-MS)

All samples were investigated by ESI-MS on an LCQ Fleet Ion Trap Mass Spectrometer (Thermo Scientific) in positive mode. All recorded MS data was analyzed in the Thermo Xcalibur Qual Browser 2.2 SP1.48 software.

HRMS (ESI) (Fmoc-AAD-OH) *m*/*z* calcd. for C_25_H_27_N_3_O_8_: 497.2; found: 497.9 [M_w_ + H]^+^, 520.2 [M_w_ + Na]^+^, 1016.6 [2M_w_ + Na]^+^ (see ESI, Fig. 26†).

HRMS (ESI) (Fmoc-AVD-OH) *m*/*z* calcd. for C_27_H_31_N_3_O_8_: 525.2; found: 525.9 [M_w_ + H]^+^, 1050.7 [2 M_w_ + H]^+^, 1072.5 [M_w_ + Na]^+^ (see ESI, Fig. 27†).

HRMS (ESI) (ac-FIID-OH) *m*/*z* calcd. for C_27_H_40_N_4_O_8_: 548.6; found: 549.3 [M_w_ + H]^+^, 571.4 [M_w_ + Na]^+^, 1118.8 [2M_w_ + Na]^+^ (see ESI, Fig. 28†).

HRMS (ESI) (Fmoc-AAC(NBD)D-OH) *m*/*z* calcd. for C_34_H_33_N_7_O_12_S: 763.7; found: 764.1 [M_w_ + H]^+^, 786.3 [M_w_ + Na]^+^, 802.1 [M_w_ + K]^+^ (see ESI, Fig. 29†).

### Cryogenic-transmission electron microscopy (cryo-TEM)

We freshly prepared samples of a 50 μL reaction volume for Cryo-TEM. Cryo-TEM imaging was operated on a Tecnai Spirit microscope (FEI/Thermo Fisher) at 120 kV. Herein, the images were recorded in a low-dose mode on a CCD camera. The samples were prepared on Cu-grids (C-flat, 2.0 μm hole size, 2.0 μm hole spacing, 400 mesh) that were freshly glow discharged for 90 seconds at 45 mA and 3·10^−2^ mbar before use. Samples were prepared as described before. 5 μL of the sample was pipetted on to the Cu-grids in a FEI/Thermo Fisher Vitrobot set to 22 °C and a relative humidity of 100%. The blotting conditions were set to a waiting time of 30 seconds, a blot time of 2.5 seconds and a blot force of −1. Thereafter, grids are plunged into liquid ethane (pre-cooled by liquid nitrogen). The cryo-TEM grids were transferred and stored in liquid nitrogen until they were placed into a Gatan cryo-transfer-specimen holder for imaging. The specimen temperature was maintained at −170 °C during the whole process. All experiments were performed in triplicate.

### UV-Vis spectroscopy

On a Multiskan FC microplate reader (Thermo Fisher), UV-Vis measurements were performed using a 96-well plate (tissue culture plate, non-treated) at 600 nm and 25 °C. All experiments were performed in triplicates.

## Data availability

All authors confirm that the data supporting the studies are available within the article and its ESI.†

## Author contributions

J. B. and M. A. W. wrote the manuscript, J. R.-F., M. A. W., B. R., A. M. B., M. K. performed the experiments, J. R.-F. and M. A. W. equally contributed to the work. J. B. and O. L. supervised the research.

## Conflicts of interest

There are no conflicts to declare.

## Supplementary Material

SC-013-D2SC03691F-s001
