# Supplementary material for: A chemically fueled supramolecular glue for self-healing gels
Source: Chem Sci. 2022 Sep 15;13(38):11411–21. doi: 10.1039/d2sc03691f (PMC9533421; doi:10.1039/d2sc03691f)
Supplement: SC-013-D2SC03691F-s001 [file SC-013-D2SC03691F-s001.pdf]

Electronic Supplementary Material (ESI) for Chemical Science.
This journal is © The Royal Society of Chemistry 2022

A chemically fueled supramolecular glue for self-healing gels†

Jennifer Rodon-Fores[1‡], Michaela A. Würbser[1‡], Martin Kretschmer[2,3], Benedikt Rieß[1], Alexander M. Bergmann[1], Oliver Lieleg[2,3], Job Boekhoven[1*]

[‡] Co-first authorship, the two authors have contributed equally to the paper.

[1] Department of Chemistry, Technical University of Munich, Lichtenbergstrasse 4, 85748 Garching, Germany.

[2] TUM School of Engineering and Design, Department for Materials Engineering, Technical University of Munich, Boltzmannstr. 15, 85748, Garching, Germany.

[3] Center for Protein Assemblies (CPA) & Munich Institute of Biomedical Engineering (MIBE), Technical University of Munich, Ernst-Otto-Fischer-Str. 8, 85748 Garching.

Supporting tables

Supporting Table S1. Characterization of synthesized peptide precursors (and commercially available benzylamine and EDC HCl).

Substance (abb.)	Structure	Mass M_w calculated [$gmol^{-1}$]	Mass found [$gmol^{-1}$]	Retention time [min]	Calibration value [$mAUmM^{-1}$] (2uL)
**Fmoc-AAD-OH**	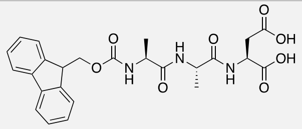	497.2<br>$C_{25}H_{27}N_3O_8$	497.9 $[M_w +H]^+$<br>520.2 $[M_w +Na]^+$<br>1016.6 $[2M_w+Na]^+$	9.7	16.65<br>(220 nm)
**Fmoc-AVD-OH**	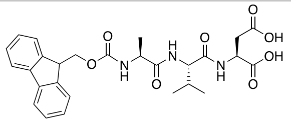	525.2<br>$C_{27}H_{31}N_3O_8$	525.9 $[M_w+H]^+$<br>1050.7 $[2M_w+H]^+$<br>1072.5 $[2M_w+Na]^+$	11.1	12.51<br>(220 nm)
**Ac-FIID-OH**	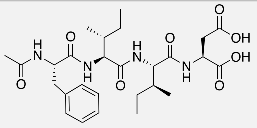	548.6<br>$C_{27}H_{40}N_4O_8$	549.3 $[M_w+H]^+$<br>571.4 $[M_w+Na]^+$<br>1118. 8 $[2M_w+Na]^+$	8.9	7.72<br>(220 nm)
**Fmoc-AAC(NBD)D-OH**	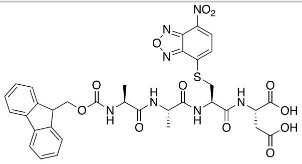	763.7<br>$C_{34}H_{33}N_7O_{12}S$	764.1 $[M_w+H]^+$<br>786.3 $[M_w+Na]^+$<br>802.1 $[M_w+K]^+$	11.7	-
**EDC· HCl**	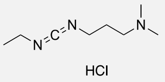	191.7<br>$C_8H_{17}N_3$<br>· HCl	-	1.38	1.75<br>(220 nm)
**BA**	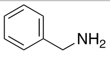	107.2<br>C_7H_9N	-	2.38	0.37<br>(220 nm)

Supporting Table S2: Summary of rate constants of the peptide precursor acids for the reaction cycle using the kinetic model.

Peptide precursor/ order of rate	k_0 [s^{-1}]	k_1 [$M^{-1}s^{-1}$]	k_2 [s^{-1}]	k_3 [s^{-1}]	k_4 [s^{-1}]	Half-life (anhydride) [s] $\tau_{1/2} = \ln(2)/k_4$
**Order**	1st	2nd	1st	1st	1st	-
**[a]Fmoc-AAD-OH**	1.35×10^{-6}	0.045	0.8	0.35	0.015	46
**[b]Fmoc-AVD-OH**	7.0×10^{-6}	0.280	2 x k_1	2.5 x k_1	0.015	46
**[c]Ac-FIID-OH**	1.35×10^{-6}	0.95×10^{-2}	2 x k_1	2 x k_1	0.75×10^{-2}	92

a. 10 mM Fmoc-AAD-OH fueled with 500 mM EDC.
b. 10 mM Fmoc-AVD-OH fueled with 100 mM EDC.[1]
c. 15 mM ac-FIID-OH fueled with 25 mM EDC.[2]

Supporting figures

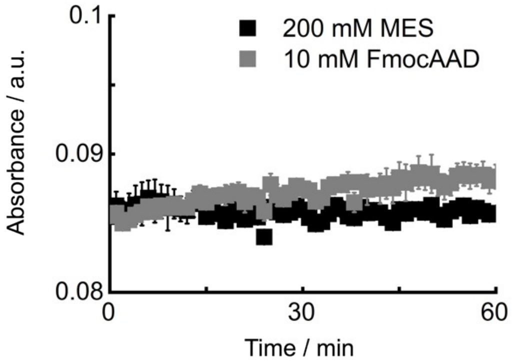

Fig. 1: UV/Vis absorbance at 600 nm over time of 200 mM MES buffer (black) and 10 mM peptide precursor acid Fmoc-AAD-OH in 200 mM MES without fuel (grey). All experiments were performed in triplicate.

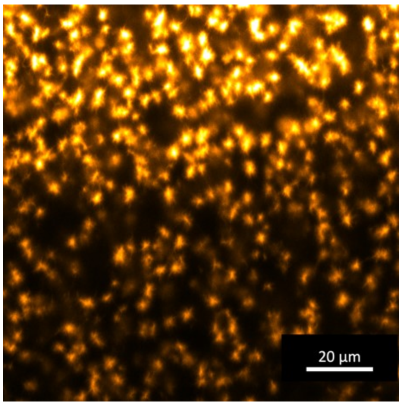

Fig. 2: Confocal fluorescence micrograph of 10 mM peptide precursor acid Fmoc-AAD-OH fueled with 500 mM EDC showing a dense network of fiber bundles.

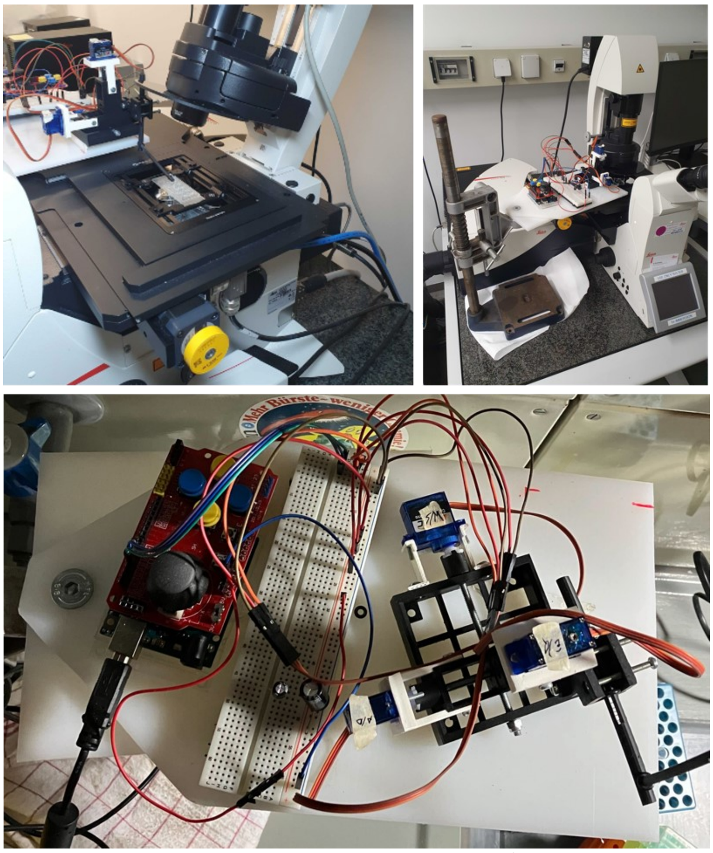

Fig. 3:Photographs of the micromanipulator while performing a scratch through a hydrogel (10 mM Fmoc-AAD-OH fueled with 500 mM EDC) (left) and the entire micromanipulator in wholesome profile view (right) and top view (bottom).

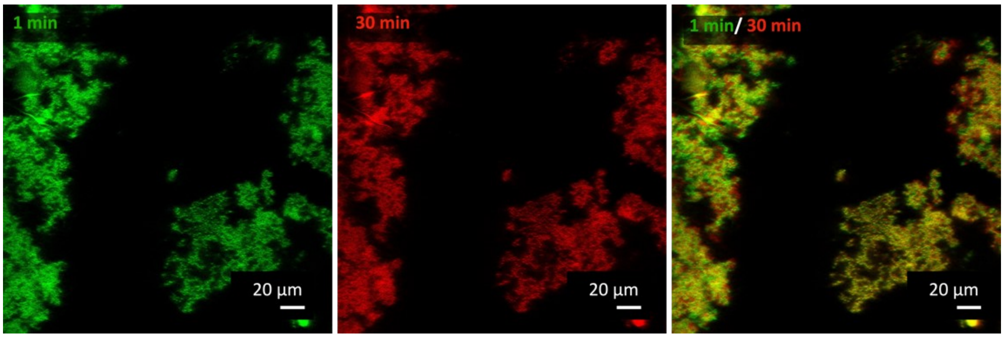

Fig. 4: Confocal micrographs of 10 mM Fmoc-AAD-OH acidified with 500 mM 1 minute (green) after performing a scratch 13 minutes after fuel addition and after 30 minutes (red). The composites showed no healing of the network after 30 minutes. The acidification took place in a faster manner compared to fueling with EDC resulting in nanofibers, but in a more agglomerated assembly structure leading to an uneven scratch. All experiments were performed in triplicate.

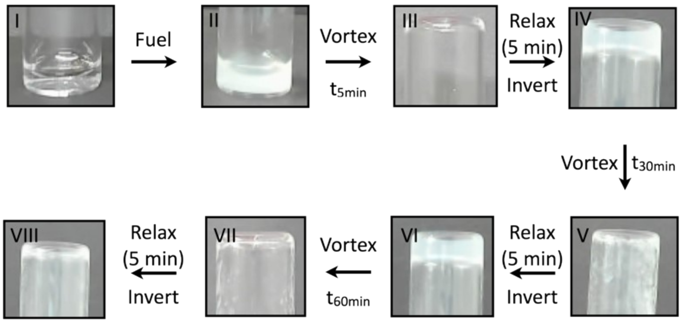

Fig. 5: Vial inversion test of 10 mM Fmoc-AAD-OH (I) fueled with 500 mM EDC (II). The hydrogel was vortex mixed for 20 seconds 5 minutes after fuel addition (III) showing the full liquification of the gel. It was then set to relax for 5 min and inverted resulting again in a self-supporting gel (IV). This procedure was repeated at the time intervals t = 30 min (V, VI) and 60 minutes (VII, VIII). All experiments were carried out in triplicate.

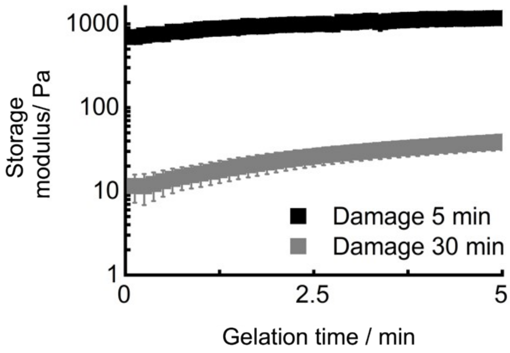

Fig. 6: Storage modulus recovery of 10 mM Fmoc-AAD-OH fueled with 500 mM EDC over a gelation time of 5 minutes after damaging the gel 5 minutes (black) and 30 minutes after fuel addition (grey). All experiments were performed in triplicate.

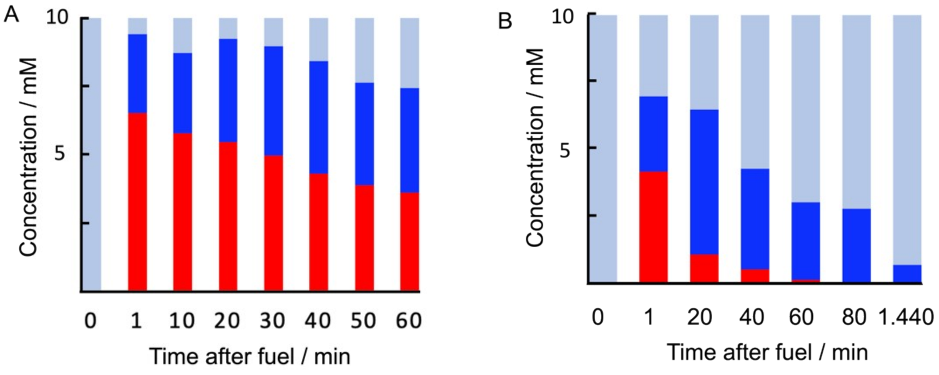

Fig. 7: Precursor remains kinetically trapped in fibers. The concentration of peptide precursor acid in the fiber assembly (blue), peptide product in the fiber assembly (red) and the peptide in solution (light blue) were determined by ^{1}H-NMR spectroscopy of 10 mM Fmoc-AAD-OH fueled with 500 mM EDC (A) or with 100 mM EDC (B). All experiments were performed in triplicate.

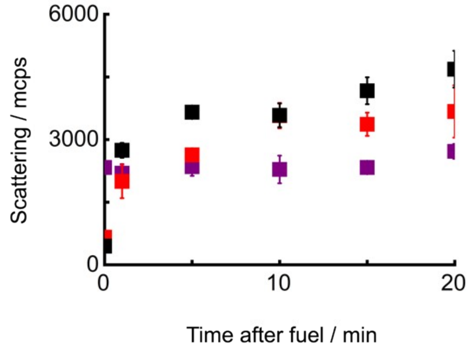

Fig. 8: Critical aggregation concentration of peptide precursor acid Fmoc-AAD-OH according to an increased scattering rate over time determined by DLS. Compared to 500 mM EDC in 200 mM MES buffer (purple), a significant increase in the scattering at t = 15 minutes was observed between 0.6 mM (red) and 0.8 mM (black) Fmoc-AAD-OH fueled with 500 mM EDC. Markers represent DLS data. The error bars show the standard deviation from the average (n = 3).

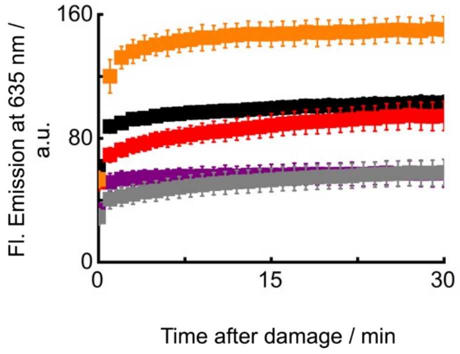

Fig. 9: NBD fluorescence intensity at 635 nm after excitation at 488 nm over time after the damage as a measure for the positive incorporation of Fmoc-AAC(NBD)D-OH into the Fmoc-AAD-OH network after an externally induced damage. A gel consisting of a mixture of 10 mM Fmoc-AAD-OH and 0.5 mM Fmoc-AAC(NBD)D-OH fueled with 500 mM EDC without damage (grey) showed a similar fluorescence emission as performing 0.5 mM Fmoc-AAC(NBD)D-OH with 500 mM EDC (aq.) with no damage (purple). In contrast, vortex mixing (red) or ultrasonication (black) of the hydrogel (10 mM Fmoc-AAD-OH fueled with 500 mM EDC) after 13 minutes and adding a mixture of Fmoc-AAD with 0.5 mM Fmoc-AAC(NBD)D-OH, led to an increased intensity (and thus to the incorporation of NBD-building blocks). The highest intensity (positive control) was achieved by directly preparing the hydrogel using 10 mM Fmoc-AAD-OH with 0.5 mM Fmoc-AAC(NBD)D-OH fueled with 500 mM EDC (orange). All markers represent experimental data in triplicate.

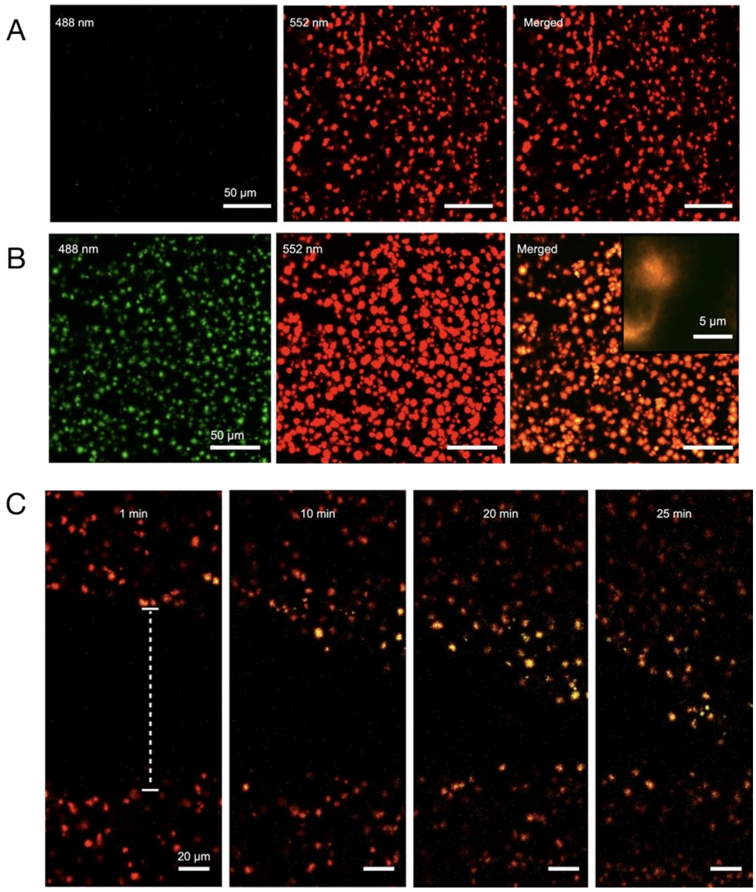

Fig. 10: Incorporation of NBD-based peptide in damaged gels. A) Confocal micrographs of the undamaged gel (10 mM Fmoc-AAD fueled with 500 mM EDC, stained with 0.1 µM Nile Red). 1 µL of a 10:1 mixture of Fmoc-AAD-OH and Fmoc-AAC(NBD)D-OH was added on top of the preformed gel. B) Confocal micrographs of the damaged gel (10 mM Fmoc-AAD fueled with 500 mM EDC, stained with 0.1 µM Nile Red). 1 µL of a 10:1 mixture of Fmoc-AAD-OH to Fmoc-AAC(NBD)D-OH was added on top of the preformed gel and damaged by ultrasonication for 2 minutes (13 minutes after fuel addition). C) Confocal micrographs of 10 mM Fmoc-AAD fueled with 500 mM EDC. After 13 minutes, a scratch was performed by applying a needle topped with 1 µL of a 10:1 mixture of Fmoc-AAD-OH and Fmoc-AAC(NBD)D-OH. The incorporation of the NBD-labeled peptide was observed over time. All experiments were carried out in triplicate.

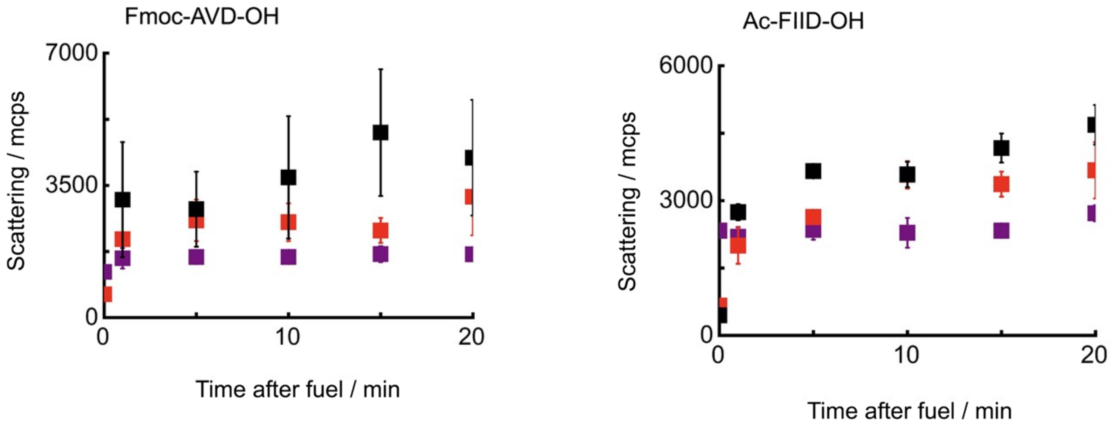

Fig. 11: Critical aggregation concentration of peptide precursor acid Fmoc-AVD-OH (left) and ac-FIID-OH (right) as measured by an increased scattering rate over time determined by DLS. Compared to 500 mM EDC in 200 mM MES buffer (purple), a significant increase in the scattering at t = 15 minutes was observed between 0.1 mM (red) and 0.3 mM (black) Fmoc-AVD-OH fueled with 500 mM EDC. Compared to 500 mM EDC in 200 mM MES buffer (purple), a significant increase in the scattering at t = 15 minutes was observed between 0.2 mM (red) and 0.3 mM (black) for ac-FIID-OH fueled with 500 mM EDC. Markers represent DLS data. The error bars show the standard deviation from the average (n = 3).

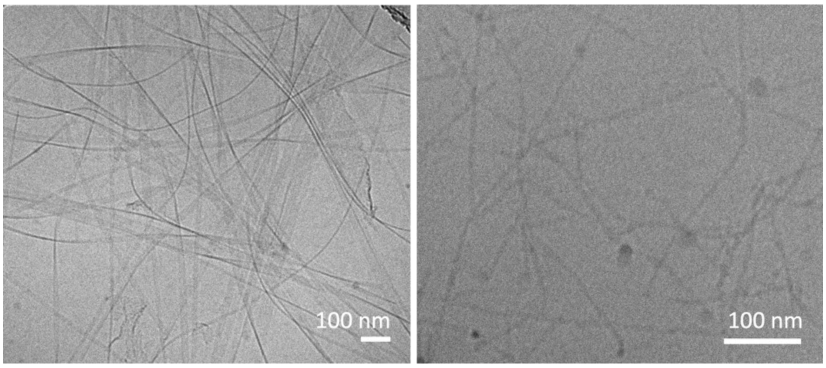

Fig. 12: Cyro-TEM micrographs of 10 mM Fmoc-AVD-OH fueled with 10 mM EDC (left) and 15 mM ac-FIID-OH fueled with 25 mM EDC (right). The micrographs were taken 1 minute after fuel addition. Both peptides formed a nanofibrillar network. All experiments were performed in triplicate.

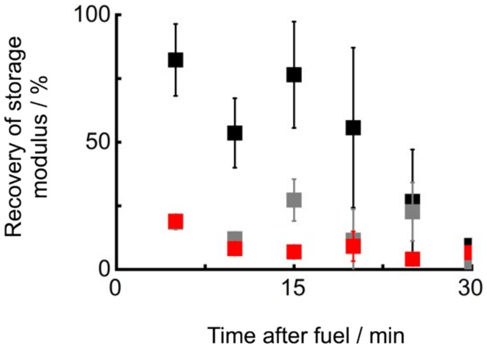

Fig. 13: Recovery of storage modulus of 10 mM peptide precursor acids Fmoc-AAD-OH (black), Fmoc-AVD-OH (grey) and ac-FIID-OH (red) fueled with 500 mM EDC damaging at various time intervals after fuel addition. Markers represent storage modulus data. The error bars show the standard deviation from the average (n = 3).

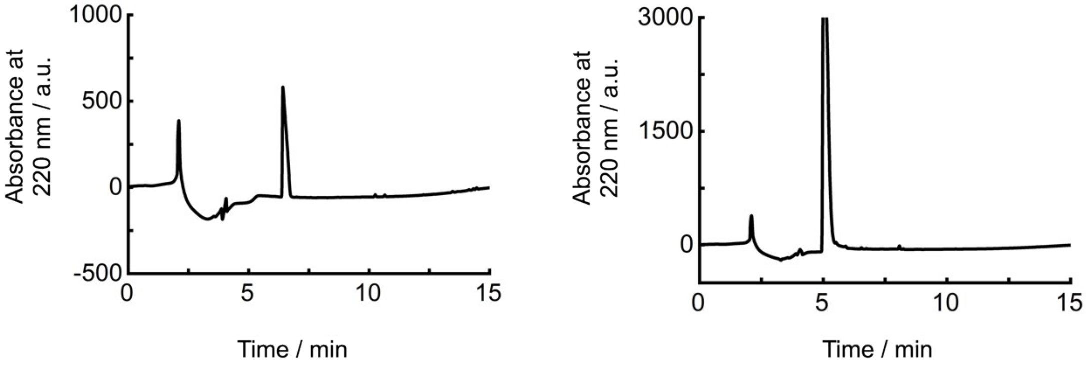

Fig. 14: HPLC chromatograms of 500 mM BA (left) and 500 mM EDC measured at 220 nm (injection volume 2 μL).

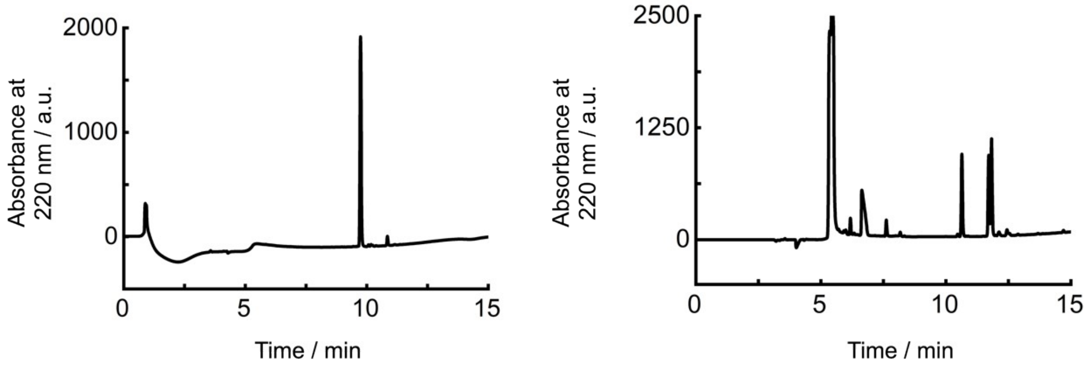

Fig. 15: HPLC chromatograms of 10 mM Fmoc-AAD-OH (left) and the entire reaction cycle 5 minutes after fuel addition (right) measured at 220 nm (injection volume 2μL).

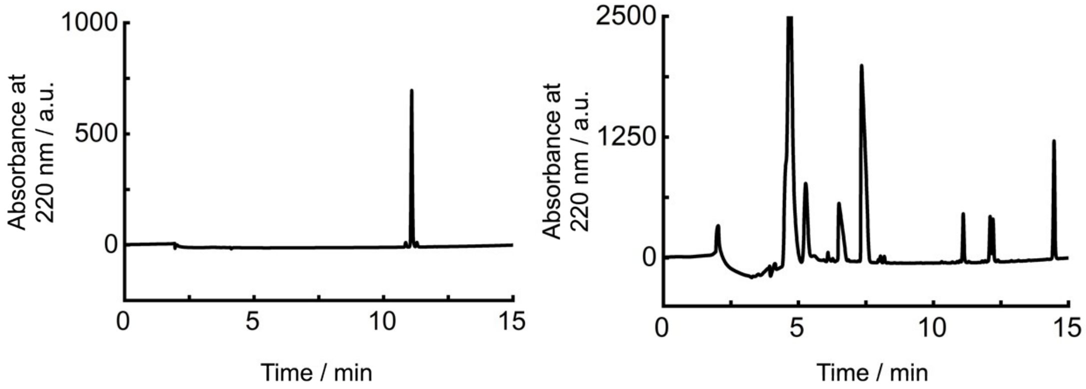

Fig. 16: HPLC chromatograms of 10 mM Fmoc-AVD-OH (left) and the entire reaction cycle 5 minutes after fuel addition (right) measured at 220 nm (injection volume 2 μL).

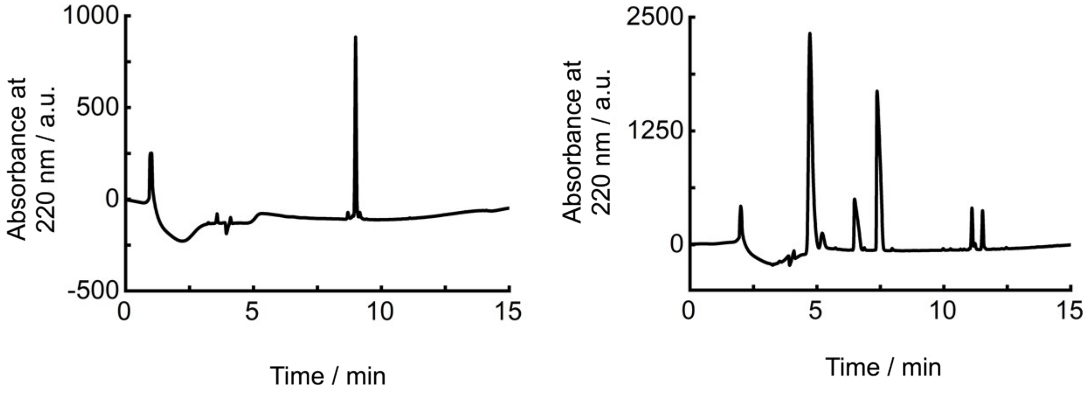

Fig. 17: HPLC chromatograms of 10 mM ac-FIID-OH (left) and the entire reaction cycle 5 minutes after fuel addition (right) measured at 220 nm (injection volume 2).

.

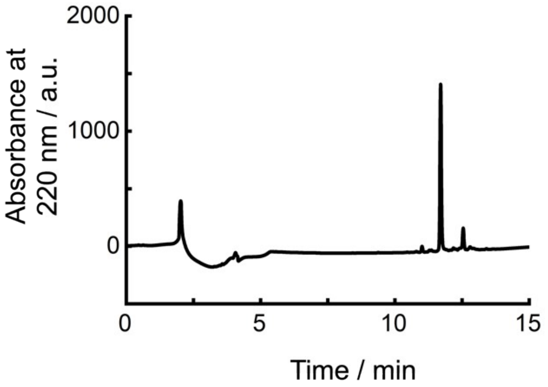

Fig. 18: HPLC chromatograms of 10 mM Fmoc-AAC(NBD)D measured at 220 nm (injection volume 2 μL).

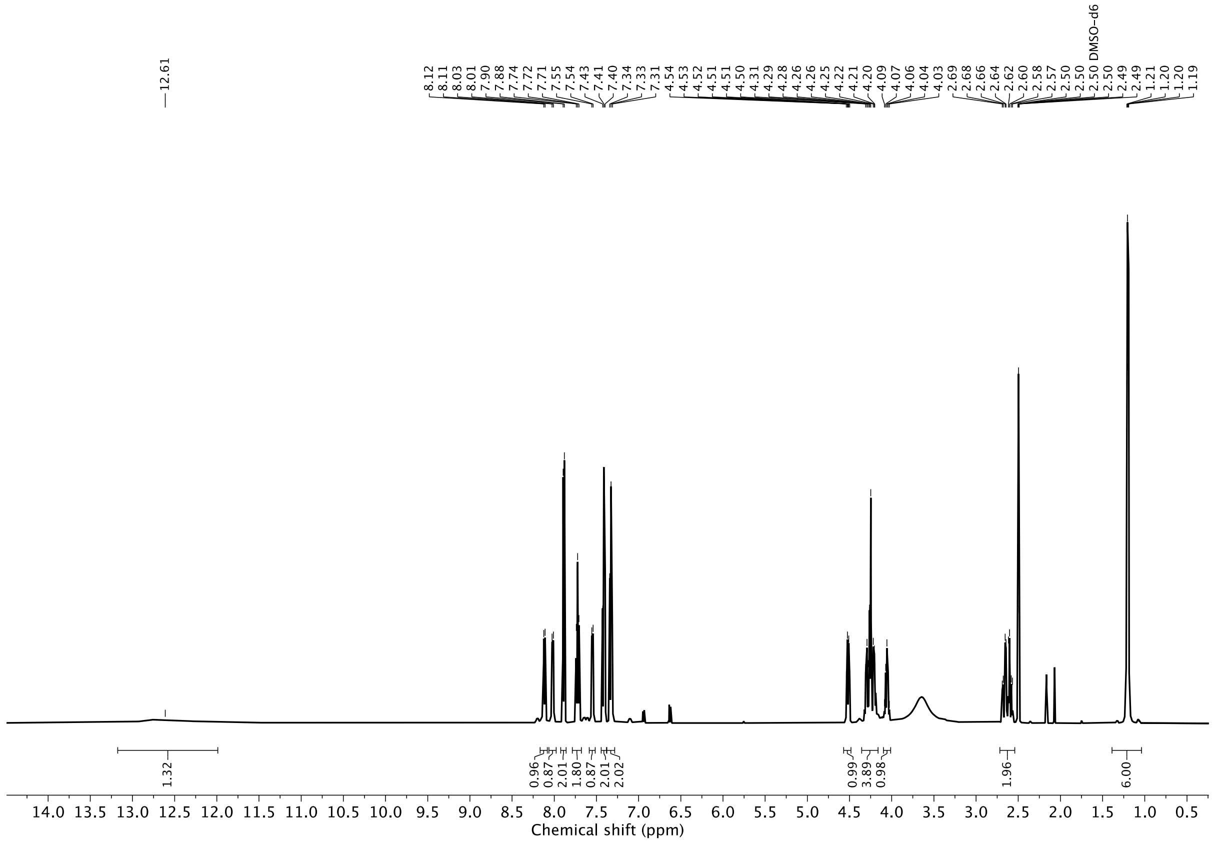

Fig. 19: ^{1}H-NMR (dmso-D$_6$, 300 MHz, 25 °C) spectrum of Fmoc-AAD-OH in dmso-D$_6$.

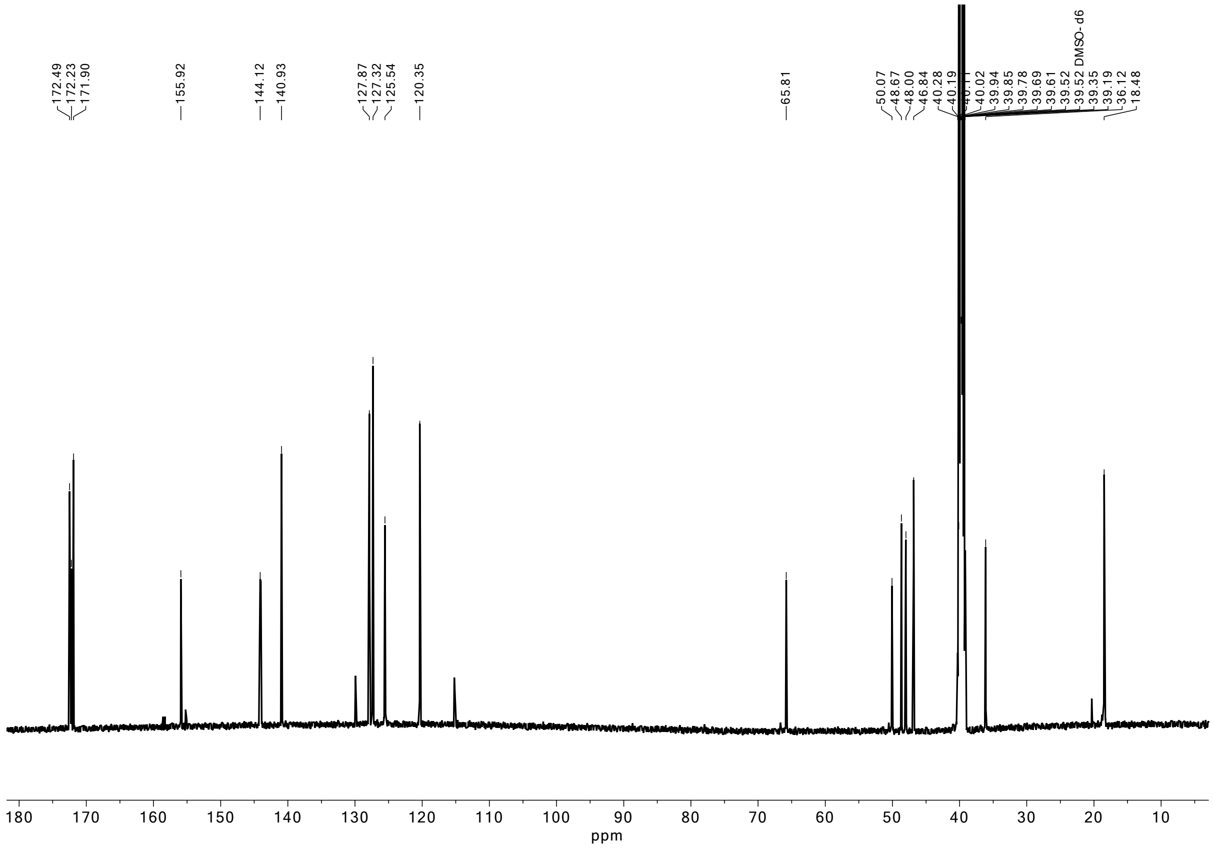

Fig. 20: ^{13}C-NMR (dmso-D$_6$, 250 MHz, 25 °C) spectrum of Fmoc-AAD-OH in dmso-D$_6$.

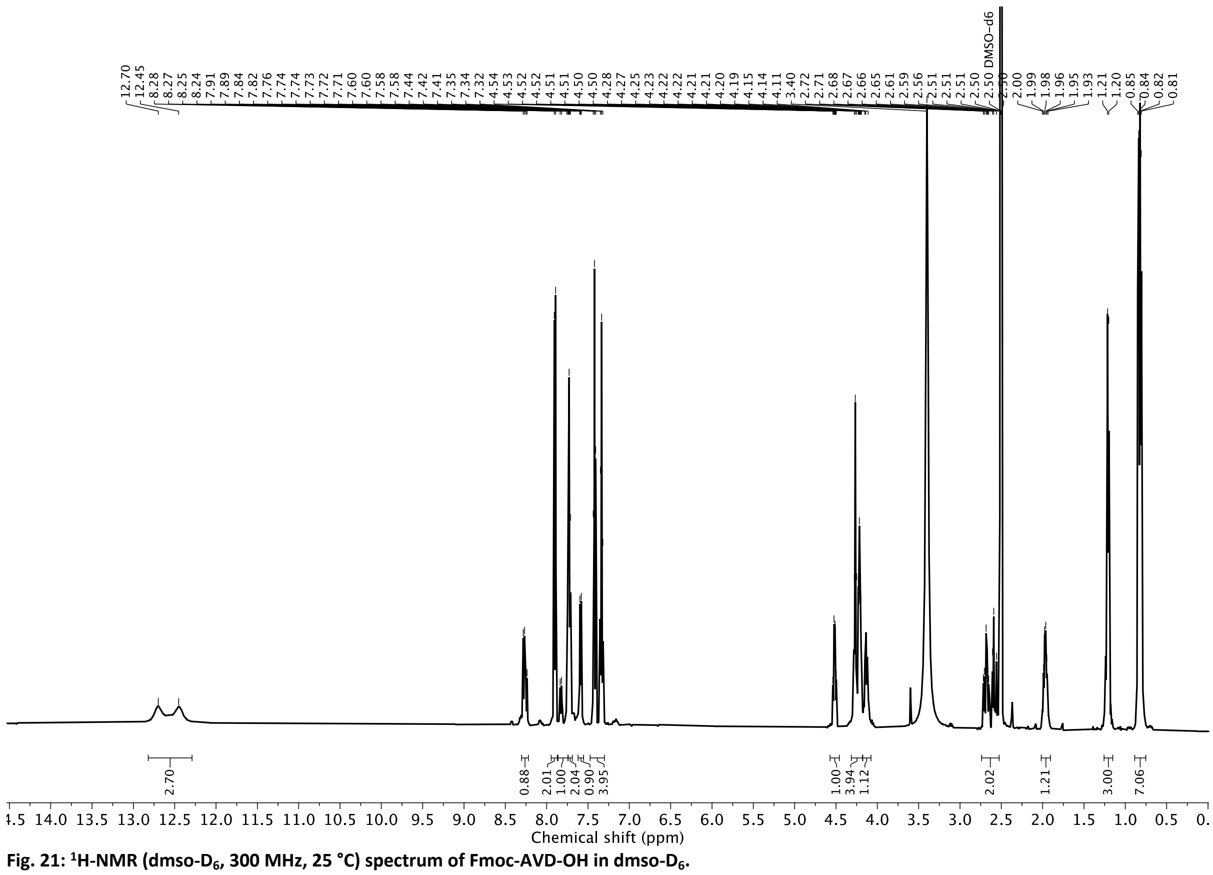

Fig. 21: ^{1}H-NMR (dmso-D$_6$, 300 MHz, 25 °C) spectrum of Fmoc-AVD-OH in dmso-D$_6$.

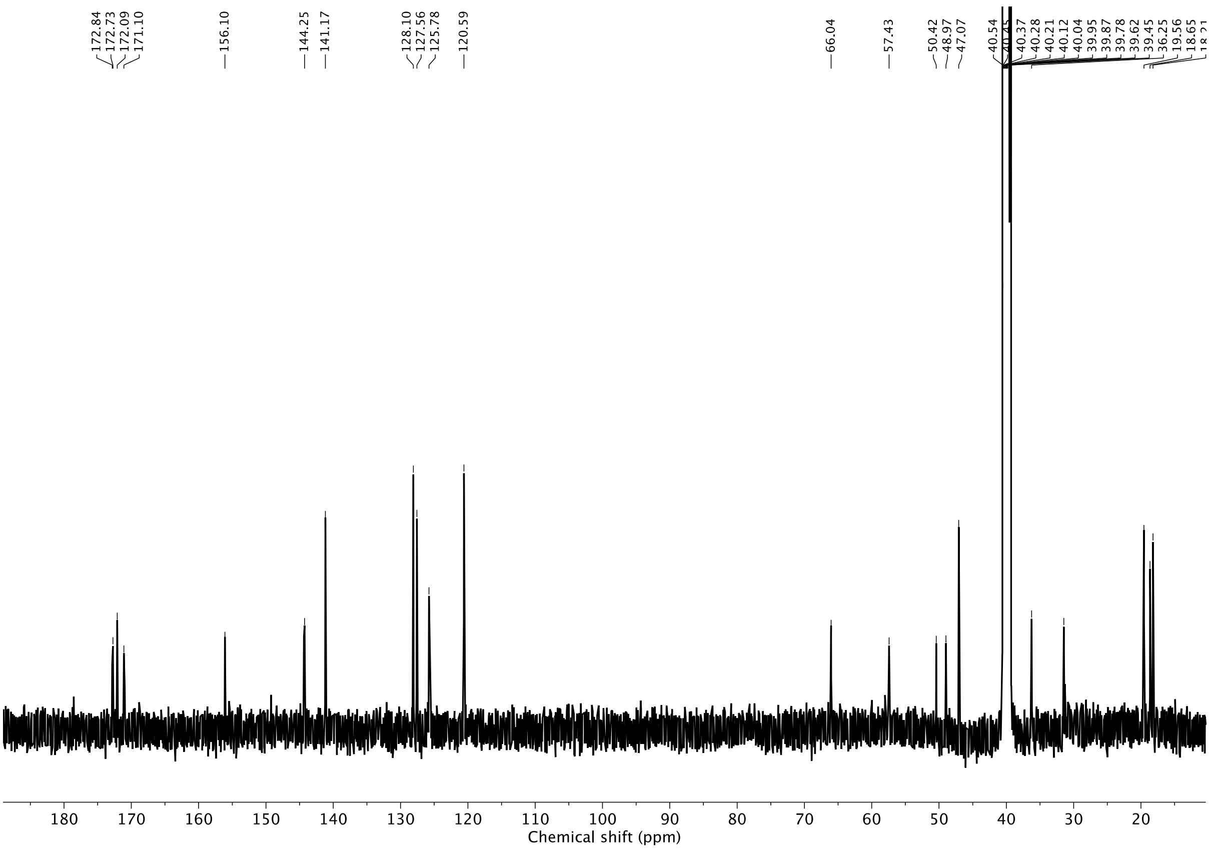

Fig. 22: ^{13}C-NMR (dmso-D$_6$, 250 MHz, 25 °C) spectrum of Fmoc-AVD-OH in dmso-D$_6$.

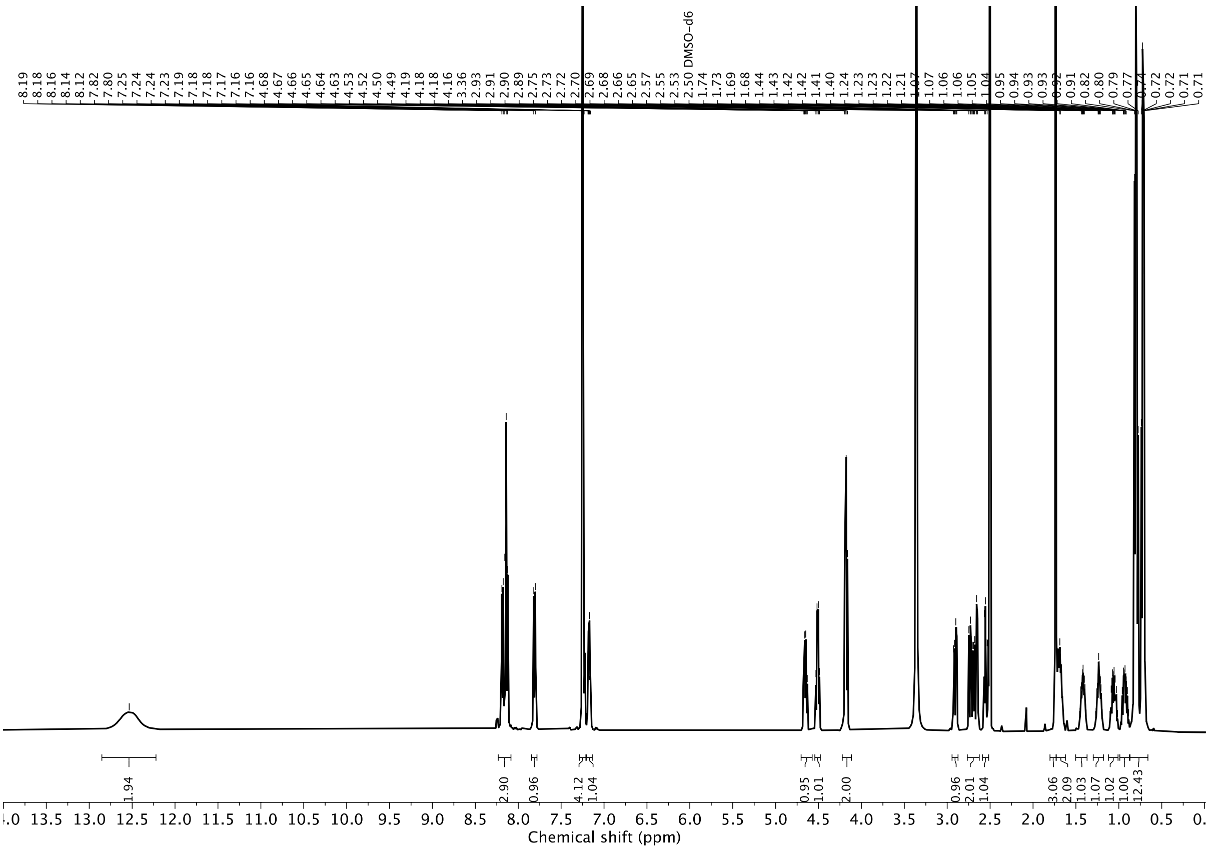

Fig. 23: ^{1}H-NMR (dmso-D$_6$, 300 MHz, 25 °C) spectrum of ac-FIID-OH in dmso-D$_6$.

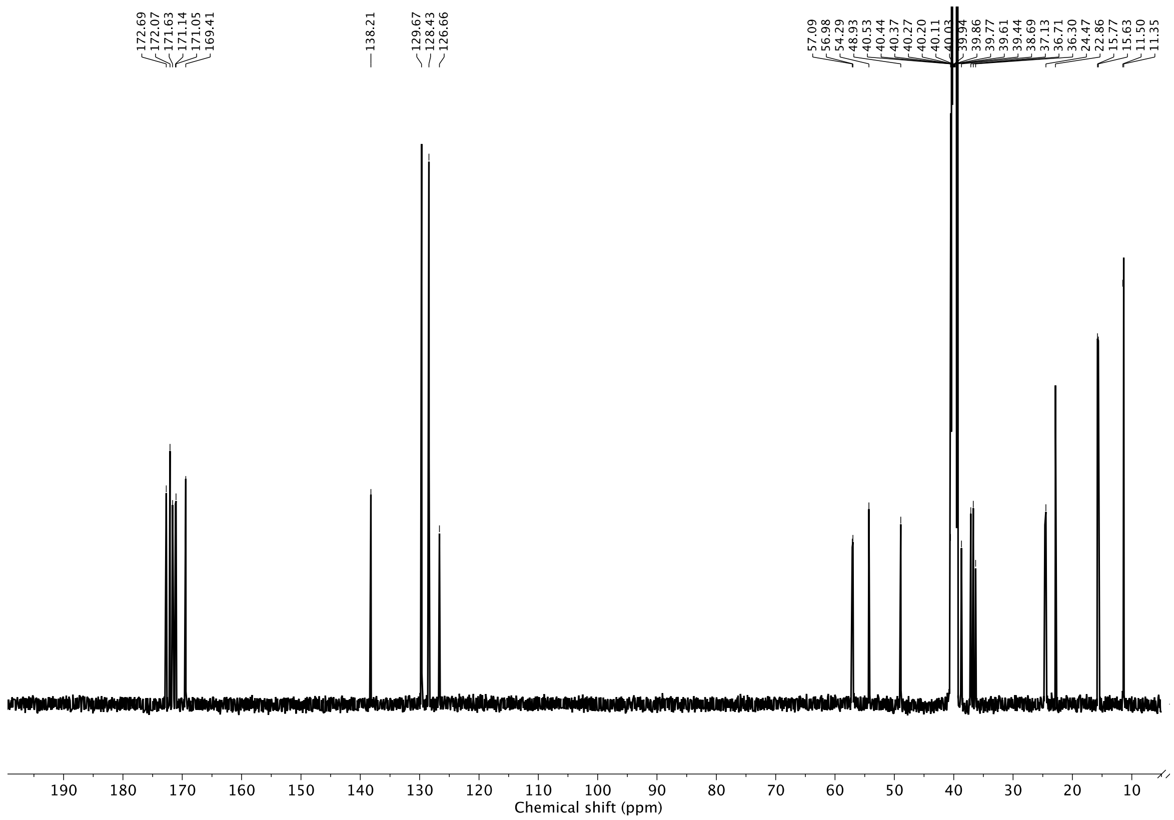

Fig. 24: ^{13}C-NMR (dmso-D$_6$, 250 MHz, 25 °C) spectrum of ac-FIID-OH in dmso-D$_6$.

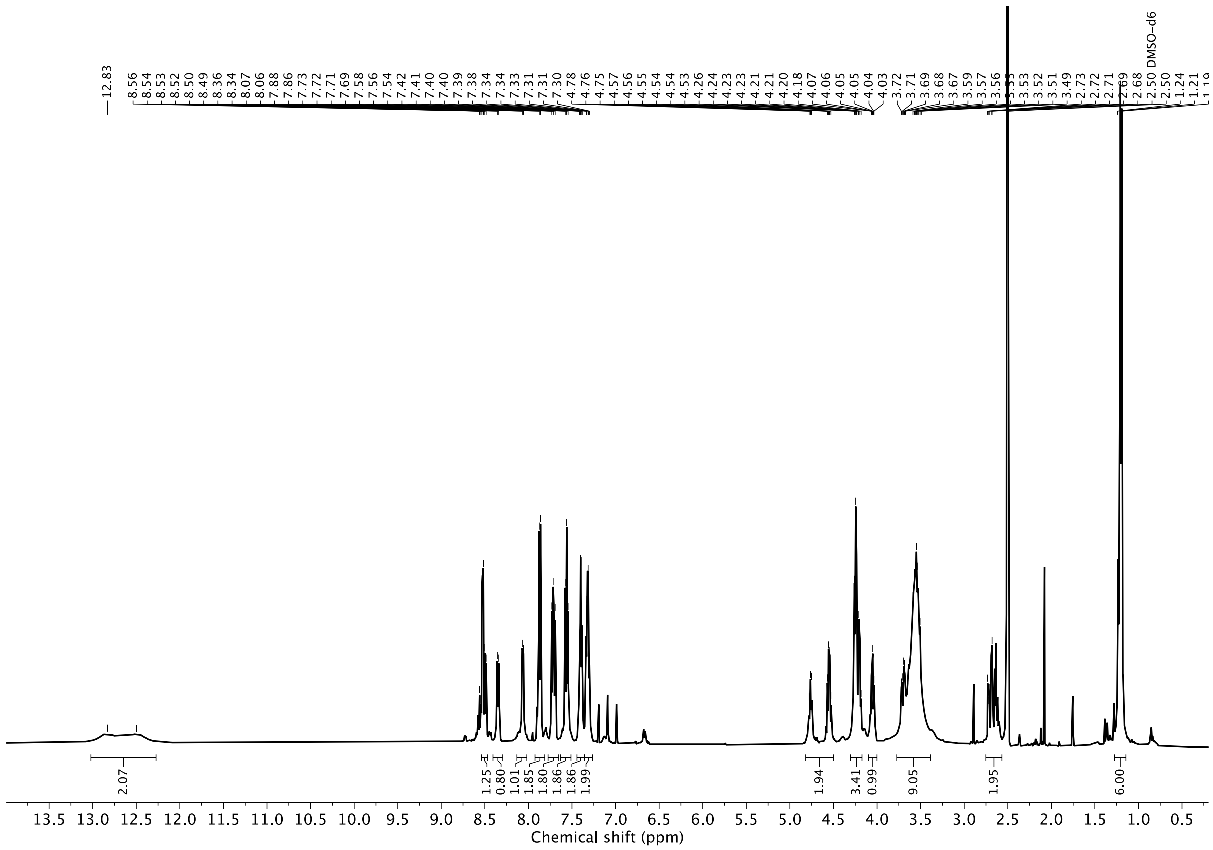

Fig. 25: ^{1}H-NMR (dmso-D$_6$, 300 MHz, 25 °C) spectrum of Fmoc-AAC(NBD)D-OH in dmso-D$_6$.

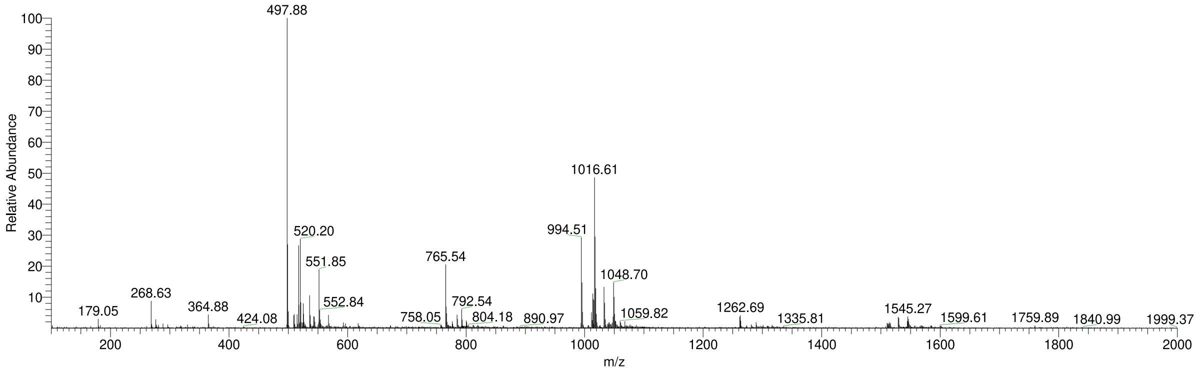

Fig. 26: ESI-MS characterization of peptide precursor acid Fmoc-AAD-OH.

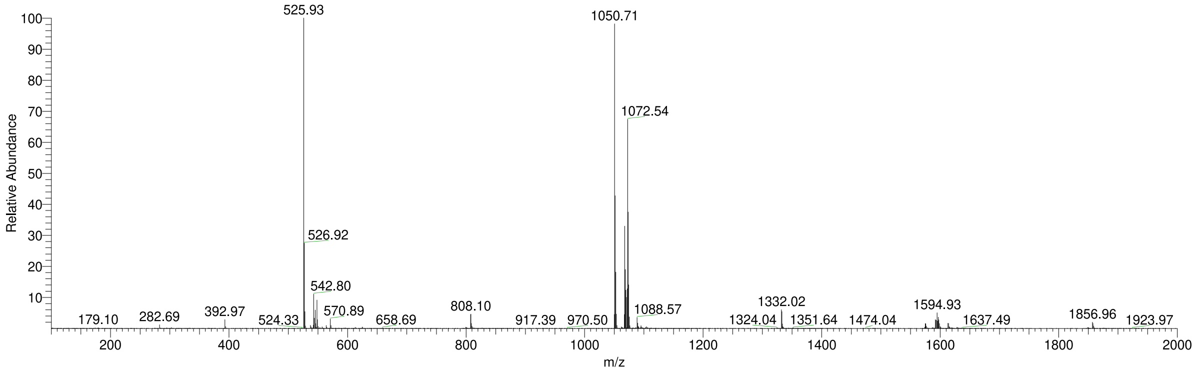

Fig. 27: ESI-MS characterization of peptide precursor acid Fmoc-AVD-OH.

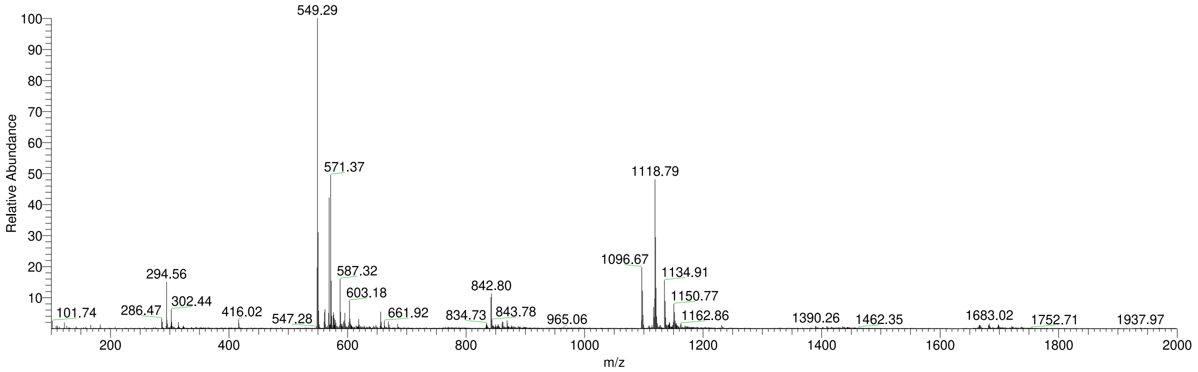

Fig. 28: ESI-MS characterization of peptide precursor acid ac-FIID-OH.

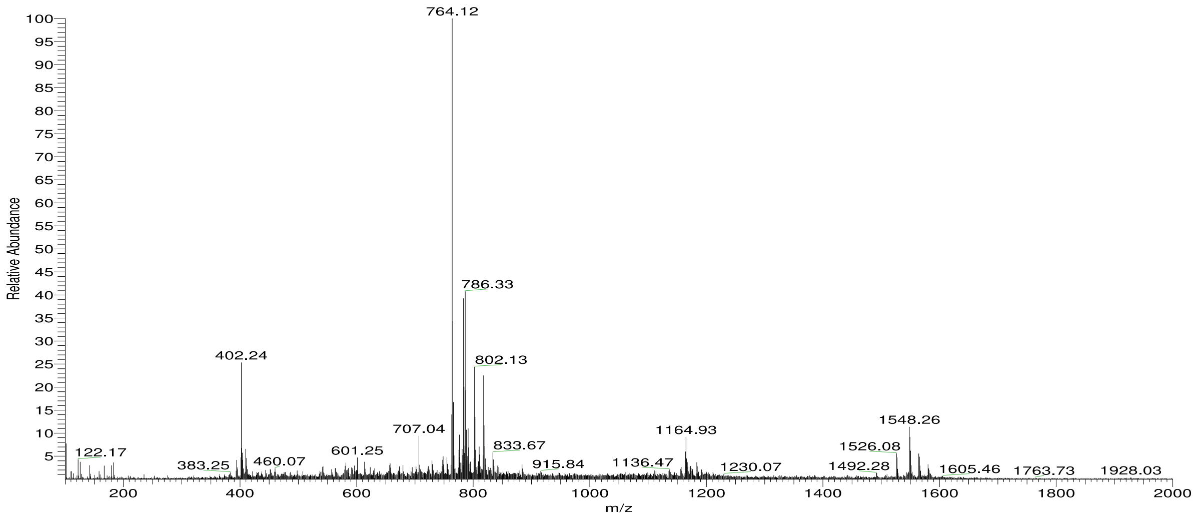

Fig. 29: ESI-MS characterization of peptide precursor acid Fmoc-AAC(NBD)D-OH.

Supporting notes

Description of the kinetic model. We used a *Matlab* code as kinetic model to calculate the concentrations of each reactant for every second *i* in the reaction cycle (peptide precursor acid, carbodiimide fuel, intermediate product, peptide product). Herein, the model consists of five differential equations which represent the five underlying chemical reactions: (1) the pseudo first-order hydrolysis reaction of EDC r_0, (2) the second-order reaction of the peptide precursor acid with the carbodiimide fuel to form the intermediate product, (3) the first-order peptide anhydride formation reaction r_2, (4) the pseudo first-order hydrolysis reaction of the intermediate product r_3, (5) the pseudo-first order hydrolysis reaction of the peptide anhydride product r_4. The rate constants k_0 and k_1 were investigated using the experimental HPLC data by monitoring the EDC concentration over time. The rate constants k_2, k_3 and k_4 were determined by fitting using the kinetic model.

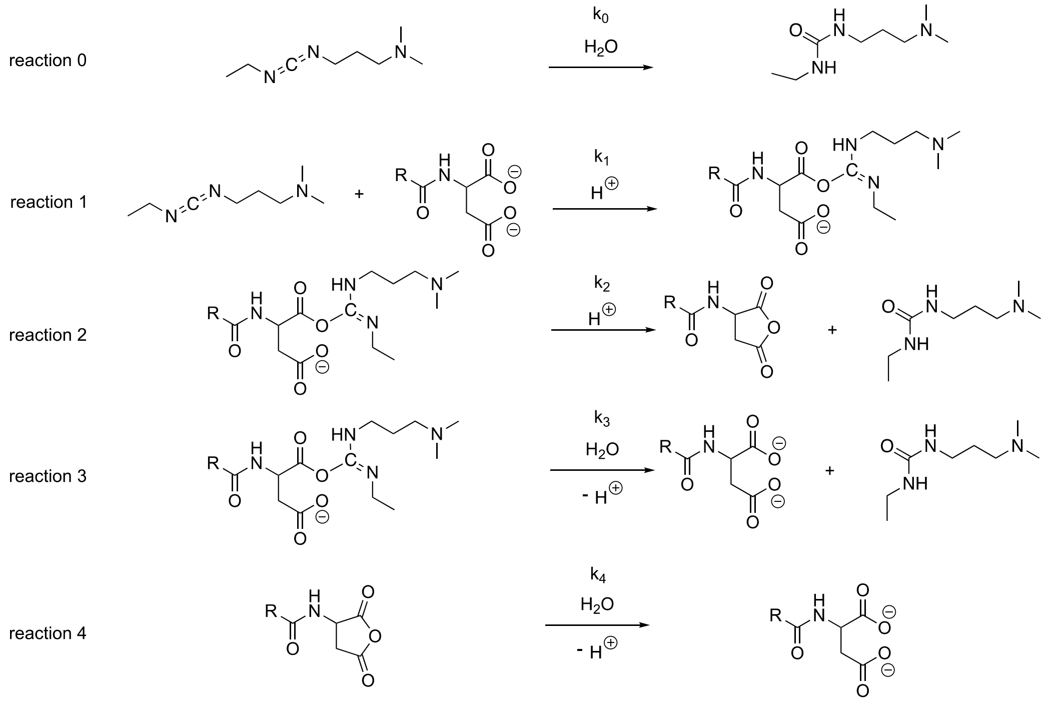

Scheme 1: Schematic representation of the chemical reactions of the kinetic model in the EDC-driven reaction cycle of peptide precursor acids. (reaction 0) Pseudo-first order hydrolysis reaction of EDC. (reaction 1) Second order reaction of the peptide precursor with EDC to form the intermediate product *O*-acylisourea. (reaction 2) First-order reaction of the intermediate product *O*-acylisourea to the anhydride product and urea waste product. (reaction 3) Pseudo first-order hydrolysis reaction of the intermediate product O-acylisourea to form the initial peptide precursor and urea waste product. (reaction 4) Pseudo-first order hydrolysis reaction of the peptide anhydride product to form the initial peptide precursor acid. See Supporting notes for a detailed discussion on the rates and rate constants.

$$r_0(i) = k_0 \cdot EDC(i) \quad (1)$$

$$r_1(i) = k_1 \cdot EDC(i) \cdot COOH(i) \quad (2)$$

$$r_2(i) = k_2 \cdot COOEDC(i) \quad (3)$$

$$r_3(i) = k_3 \cdot COOEDC(i) \quad (4)$$

$$r_4(i) = k_4 \cdot COOOC(i), \quad (5)$$

References

1. B. A. K. Kriebisch, A. Jussupow, A. M. Bergmann, F. Kohler, H. Dietz, V. R. I. Kaila and J. Boekhoven, *J. Am. Chem. Soc.*, 2020, **142**, 20837-20844.
2. K. Dai, J. R. Fores, C. Wanzke, B. Winkeljann, A. M. Bergmann, O. Lieleg and J. Boekhoven, *J. Am. Chem. Soc.*, 2020, **142**, 14142-14149.
